# Trajectories of Health Care Contact Days for Patients With Stage IV Non–Small Cell Lung Cancer

**DOI:** 10.1001/jamanetworkopen.2024.4278

**Published:** 2024-04-08

**Authors:** Arjun Gupta, Paul Nguyen, Danielle Kain, Andrew G. Robinson, Amit A. Kulkarni, David H. Johnson, Carolyn J. Presley, Anne H. Blaes, Gabrielle B. Rocque, Ishani Ganguli, Christopher M. Booth, Timothy P. Hanna

**Affiliations:** 1 Division of Hematology, Oncology, and Transplantation, University of Minnesota, Minneapolis; 2 ICES Queen’s, Queen’s University, Kingston, Ontario, Canada; 3 Division of Palliative Medicine, Department of Medicine, Queen’s University, Kingston, Ontario, Canada; 4 Division of Cancer Care and Epidemiology, Cancer Research Institute at Queens University, Kingston, Ontario, Canada; 5 Department of Oncology, Queen’s University, Kingston, Ontario, Canada; 6 Department of Internal Medicine, University of Texas Southwestern Medical Center, Dallas; 7 Division of Medical Oncology, Department of Medicine, Ohio State University, Columbus; 8 Division of Hematology and Oncology, University of Alabama at Birmingham; 9 Division of General Internal Medicine and Primary Care, Brigham and Women’s Hospital, Boston, Massachusetts

## Abstract

**Importance:**

Patients with stage IV non–small cell lung cancer (NSCLC) experience substantial morbidity and mortality. Contact days (ie, the number of days with health care contact outside the home) measure how much of a person’s life is consumed by health care, yet little is known about patterns of contact days for patients with NSCLC.

**Objective:**

To describe the trajectories of contact days in patients with stage IV NSCLC and how trajectories vary by receipt of cancer-directed treatment in routine practice.

**Design, Setting, and Participants:**

A retrospective, population-based decedent cohort study was conducted in Ontario, Canada. Participants included adults aged 20 years or older who were diagnosed with stage IV NSCLC (January 1, 2014, to December 31, 2017) and died (January 1, 2014, to December 31, 2019); there was a maximum 2-year follow-up. Data analysis was conducted from February 22 to August 16, 2023.

**Exposure:**

Systemic cancer-directed therapy (yes or no) and type of therapy (chemotherapy vs immunotherapy vs targeted therapy).

**Main Outcomes and Measures:**

Contact days (days with health care contact, outpatient or institution-based, outside the home) were identified through administrative data. The weekly percentage of contact days and fitted models with cubic splines were quantified to describe trajectories from diagnosis until death.

**Results:**

A total of 5785 decedents with stage IV NSCLC were included (median age, 70 [IQR 62-77] years; 3108 [53.7%] were male, and 1985 [34.3%] received systemic therapy). The median overall survival was 108 (IQR, 49-426) days, median contact days were 36 (IQR, 21-62), and the median percentage that were contact days was 33.3%. A median of 5 (IQR, 2-10) days were spent with specialty palliative care. Patients who did not receive systemic therapy had a median overall survival of 66 (IQR, 34-130) days and median contact days of 28 (IQR, 17-44), of which a median of 5 (IQR, 2-9) days were spent with specialty palliative care. Overall and for subgroups, normalized trajectories followed a U-shaped distribution: contact days were most frequent immediately after diagnosis and before death. Patients who received targeted therapy had the lowest contact day rate during the trough (10.6%; vs immunotherapy, 15.4%; vs chemotherapy, 17.7%).

**Conclusions and Relevance:**

In this cohort study, decedents with stage IV NSCLC had a median survival in the order of 3.5 months and spent 1 in every 3 days alive interacting with the health care system outside the home. These results highlight the need to better support patients and care partners, benchmark appropriateness, and improve care delivery.

## Key Points

**Question:**

What are the trajectories of contact days (days with health care contact outside the home) for patients with stage IV non–small cell lung cancer (NSCLC)?

**Findings:**

In this cohort study including 5785 decedents with stage IV NSCLC, the median survival was 3.5 months and patients had spent 1 in 3 of those days with health care contact outside the home. Normalized trajectories followed a U-shaped distribution such that contact days were most frequent immediately after diagnosis and immediately before death, with a middle trough.

**Meaning:**

The study findings suggest that patients' and their care partners' lives may be consumed by health care, and there is a need to benchmark appropriateness, better support patients and care partners, and improve care delivery.

## Teaser

This cohort study of decedents with stage IV non–small cell lung cancer estimates the time individuals spent interacting with the health care system outside their homes during the period between diagnosis and death.

## Introduction

Patients with lung cancer face major morbidity and mortality and frequently interact with the health care system due to the cancer, treatment, and comorbidities.^1,2,3,4,5,6^ These frequent health care interactions, although sometimes essential, can take personal time away from patients facing limited life expectancy. The time losses faced by patients have recently been conceptualized as the time toxicity of treatment, and the oncology discipline has been urged to measure, report, and improve these time burdens.^7,8,9,10^ Health care contact days—days spent receiving health care outside the home—is a patient-centered, practical, and intuitive construct to measure time toxicity.^7,11,12,13,14^

There is early evidence that patients with advanced cancers spend a large share of their days as health care contact days. For example, patients with advanced gastrointestinal cancer, with a median survival of 6 months, spend 1 in 4 days during this period receiving health care.^6,15^ Over the course of their illnesses, these contact days follow a U-shaped trajectory, with an initial peak, a middle trough, and a peak again as patients approach the end of life.^6,15^ However, analyses have been limited to single centers with a limited variety of treatments. Analyzing patterns of contact days in a large population-based cohort in a primary cancer site with different treatment approaches, including novel therapies purported to reduce patient burdens, would be informative to decision-makers, clinicians, and patients.

Given the incidence, morbidity, and mortality burden of lung cancer and specifically, stage IV non–small cell lung cancer (NSCLC),^5^ and availability of several new systemic cancer–directed treatments over the past decade,^16^ we sought to characterize contact days among patients with stage IV NSCLC in a large population-based cohort. We specifically sought to examine trajectories of contact days by receipt and specific types of systemic cancer-directed treatment in routine practice.

## Methods

### Setting and Case Selection

We created a population-based, retrospective cohort of adult patients (aged 20 years or older at diagnosis) diagnosed with stage IV NSCLC using administrative data collected by the Ministry of Health covering the population of Ontario, Canada. Ontario is the largest Canadian province with a population of 15.6 million, approximately 39% of the total Canadian population, and provides universal health care coverage to all eligible residents.^17^ We included patients with stage IV NSCLC from the Ontario Cancer Registry (eTable 1 in Supplement 1) who were diagnosed from January 1, 2014, to December 31, 2017, and died from January 1, 2014, to December 31, 2019. This strategy allowed for adequate follow-up (maximum, 2 years) and sample size, while avoiding years of disruption due to the COVID-19 pandemic. Additionally, several new drugs were used during this time period (eTable 2 in Supplement 1).^18^ Stage at diagnosis was based on American Joint Committee on Cancer 7th and 8th editions. The study was approved by the Queen’s University Health Sciences and Affiliated Teaching Hospitals Research Ethics Board and followed the Strengthening the Reporting of Observational Studies in Epidemiology (STROBE) and Reporting of studies Conducted using Observational Routinely-collected Data (RECORD) guidelines. A waiver of informed consent was granted based on the Personal Health Information Protection Act, Section 44(1).

### Data Sources and Linkage

Data were obtained from administrative data sets housed at ICES (formerly known as the Institute for Clinical Evaluative Sciences), which is an independent, nonprofit research institute funded by an annual grant from the Ontario Ministry of Health and Long-Term Care. Cancer-specific data were abstracted from the Ontario Cancer Registry, a population-based tumor registry administered by Ontario Health. The registry passively collects cancer data on Ontario residents through pathology reporting, hospital records, treatment centers, and death records. Demographic information was abstracted from the Registered Persons Database, a repository for residents of Ontario who are eligible for the Ontario Health Insurance Plan. Health use data were abstracted from multiple administrative databases listed and are described in eTable 3 in Supplement 1. These data sets were linked using unique encoded identifiers and analyzed at ICES.

### Covariates

We extracted sociodemographic and clinical characteristics using multiple databases (eTable 3 in Supplement 1). These covariates were used in multivariable adjusted analyses. Characteristics included age, sex, income, place of residence, rurality of residence, comorbidities, year of cancer diagnosis, cancer anatomical location, and histologic and morphologic factors. Comorbidities were measured using the Elixhauser comorbidity index derived from hospital records with a 5-year look back from their NSCLC diagnosis. Chronic conditions (eg, asthma, hypertension, and dementia) were based on the ICES-derived databases. Area-level age-sex standardized smoking status data were available for all individuals. For the proximity from place of residence to the nearest regional cancer center (obtained from Ontario Health), the shortest driving distance and duration were estimated using the Open Source Routing Machine API with OpenStreetMap.^19^ Cancer-directed treatment, including receipt of systemic therapy, radiotherapy, or metastasis surgery from NSCLC diagnosis to death were identified. Prior health care use, including inpatient admissions and emergency department visits with a 1-year lookback from NSCLC, was extracted. Patient-level symptoms and performance status were assessed with the Edmonton Symptom Assessment System with a 3-month period from their NSCLC diagnosis. These symptoms were classified into 3 clusters: localized physical (pain, nausea, and shortness of breath), generalized physical (tiredness, drowsiness, lack of appetite, and low well-being), and mood-based (anxiety, and depression) symptoms.

### Exposures and Outcomes

The primary exposure was receipt of systemic cancer–directed therapy (yes vs no), described in eTable 2 in Supplement 1. Among patients who received any systemic therapy, the type of systemic therapy received was categorized into 3 subgroups: all lines of cytotoxic chemotherapy only (cytotoxic, clinical trial, or other multiagent systemic therapy), first or subsequent line of immunotherapy (immunotherapy with or without chemotherapy), and first or subsequent line of targeted therapy. These patients were also classified by those who received only 1 line and 2 lines of therapy in the metastatic setting (patients with ≥3 lines of therapy were not specifically described due to inadequate sample sizes) and time to initiation of systemic therapy from diagnosis.

The primary outcome was health care contact days (from stage IV NSCLC diagnosis to death), defined as health care contact outside the home, regardless of the duration, cause, or location of contact on that day. We considered days without health care contact outside the home as home days (received no health care, received virtual care, or received home care visits). Data were abstracted from administrative databases listed in eTable 3 in Supplement 1.

Health care contact days were classified into 2 subgroups: institution based (inpatient acute or rehabilitation hospitalizations, emergency department visits, or long-term or complex continuing care) and outpatient (eg, family physician and cancer clinic visits, blood tests, imaging, outpatient surgeries, dialysis, injections/infusions, and radiotherapy assessment and treatments). The following blood tests were included: red blood cell, white blood cell, platelet, bilirubin, potassium, thyroid stimulating hormone, creatinine, or hemoglobin A_1c_. If institution-based and outpatient care occurred on the same day, it was only considered as an institution-based contact. We additionally extracted and summarized days with specialty palliative care and days with radiation oncology care (visit with a radiation oncology clinician or radiation therapy treatment).

Overall survival, which is the sum of health care contact days and home days, was measured from date of stage IV NSCLC diagnosis to death. Vital status was censored 2 years from diagnosis, with the latest date of follow-up being December 31, 2019.

### Statistical Analysis

We generated summary statistics for sociodemographic and clinical characteristics. Results were stratified by receipt of systemic therapy (yes vs no), survival duration (≤6 vs >6 months), and time to initiation of systemic therapy. Median percentage of health care contact days was estimated as median number of contact days divided by median overall survival.

We plotted the percentage of weekly contact days (percentage of contact days in each week) from diagnosis to death. To facilitate visualization of trajectories of percentage of contact days over time across patients with differential survival, we rescaled (minimum-maximum normalization) the time from diagnosis to death and fitted a cubic smoothing spline to the normalized observations. Plots were also created for the percentages of weekly institution-based and outpatient contact days. We generated these trajectory plots for all the exposure subgroups. Among patients receiving specific lines of therapy, we divided the normalized time into phases: pretreatment, during treatment, intertreatment, and posttreatment, by the mean time spent in each phase.

To further compare patients who did and did not receive systemic therapy, we used modified Poisson regression to model contact days in the first month after diagnosis, month with the lowest contact days, and the last month before death with sociodemographic and clinical characteristics. We hypothesized that different factors would be associated with a higher number of contact days during different phases of care. Statistical significance was determined with 2-sided testing and a threshold of *P* < .05. We conducted analyses from February 22 to August 16, 2023, using SAS software, version 9.4 (SAS Institute LLC).

## Results

We included 5785 patients with stage IV NSCLC (eFigure 1 in Supplement 1). Detailed sociodemographic and clinical characteristics are presented in the Table. These patients had a median age of 70 (IQR, 62-77) years, 2677 were female (46.3%), and 3108 were male (53.7%). The most common histologic type of cancer was adenocarcinoma (57.8%). Of the total cohort, 3800 patients (65.7%) did not receive systemic cancer-directed therapy. Patients who did not receive systemic treatment were older (median age, 72 [IQR, 64-79] vs 66 [IQR, 60-72] years; ≥80 years, 24.5% vs 6.5%), and the cancers were more likely to have a squamous histologic characteristic (19.7% vs 12.9%).

**Table.** Sociodemographic and Clinical Characteristics of Patients Diagnosed With Stage IV Non–Small Cell Lung Cancer

Characteristic	Patients, No. (%)^a^		
	Total (N = 5785)	Systemic therapy	
		Yes (n = 1985)	No (n = 3800)
Sociodemographic			
Age, y			
Median (IQR)	70 (62-77)	66 (60-72)	72 (64-79)
20-59	1011 (17.5)	495 (24.9)	516 (13.6)
60-69	1845 (31.9)	795 (40.1)	1050 (27.6)
70-79	1870 (32.3)	566 (28.5)	1304 (34.3)
≥80	1059 (18.3)	129 (6.5)	930 (24.5)
Sex			
Female	2677 (46.3)	975 (49.1)	1702 (44.8)
Male	3108 (53.7)	1010 (50.9)	2098 (55.2)
Income quintile			
1 (lowest)	1502 (26.0)	423 (21.3)	1079 (28.4)
2	1331 (23.0)	452 (22.8)	879 (23.1)
3	1061 (18.3)	377 (19.0)	684 (18.0)
4	993 (17.2)	377 (19.0)	616 (16.2)
5 (highest)	877 (15.2)	351 (17.7)	526 (13.8)
Urban/rural residence			
Urban (RIO<10)	3643 (63.0)	1223 (61.6)	2420 (63.7)
Suburban (10≤RIO<40)	1458 (25.2)	518 (26.1)	940 (24.7)
Rural (RIO≥40)	595 (10.3)	207 (10.4)	388 (10.2)
Chronic conditions			
Asthma	771 (13.3)	238 (12.0)	533 (14.0)
COPD	2433 (42.1)	705 (35.5)	1728 (45.5)
Hypertension	3438 (59.4)	1028 (51.8)	2410 (63.4)
CHF	554 (9.6)	121 (6.1)	433 (11.4)
Dementia	179 (3.1)	14 (0.7)	165 (4.3)
CKD^b^	554 (9.6)	94 (4.7)	460 (12.1)
Clinical			
Histologic/morphologic status			
Neoplasms, NOS	1264 (21.8)	386 (19.45)	878 (23.1)
Squamous cell neoplasms	1003 (17.3)	256 (12.9)	747 (19.7)
Adenomas or adenocarcinomas	3341 (57.8)	1270 (64.0)	2071 (54.5)
Other	177 (3.1)	73 (3.7)	104 (2.7)
ESAS assessment^c^			
No. of assessments			
No. (%)	3451 (59.7)	1723 (86.8)	1728 (45.5)
Median (IQR)	3 (1-5)	4 (2-5)	2 (1-3)
Localized physical symptom score, median (IQR)	7 (4-8)	6 (4-8)	7 (4-9)
Generalized physical symptom score, median (IQR)	8 (5-9)	7 (5-9)	8 (6-9)
Mood-based symptom score, median (IQR)	5 (2-7)	5 (2-7)	5 (2-8)
Nearest cancer center from place of residence^d^			
Estimated shortest driving distance, median (IQR), km	22 (8-56)	23 (9-57)	21 (8- 55)
Estimated shortest driving duration, median (IQR), min	25 (14-47)	26 (15-48)	24 (14- 46)

Abbreviations: CHF, congestive heart failure; CKD, chronic kidney disease; COPD, chronic obstructive pulmonary disease; ESAS, Edmonton Symptom Assessment System; NOS, not otherwise specified; RIO, Rurality Index for Ontario.

^a^ Column percentages may not sum to 100% due to missing data.

^b^ Chronic kidney disease was measured with an average estimated glomerular filtration rate of less than 60 mL/min/1.73 m^2^ from multiple laboratory tests within a 1-year look back period from non–small cell lung cancer (NSCLC) diagnosis. We lacked laboratory data from 1 of the 14 health regions, accounting for 14.3% of the study cohort.

^c^ The ESAS assessments were measured within a 3-month look back and look forward periods from NSCLC diagnosis; ESAS symptoms were categorized as localized physical (pain, nausea, and shortness of breath), generalized physical (tiredness, drowsiness, lack of appetite, and well-being) and mood-based symptom clusters (anxiety and depression); maximum intensity scores from any ESAS symptom within the cluster were used when multiple assessments were reported.

^d^ Driving distance and duration were measured with the shortest distance generated from the Open Source Routing Machine API with OpenStreetMap data between the postal code of residence and the geographic location of the regional cancer center.

For the whole cohort, median overall survival was 108 (IQR, 49-426) days and health care contact days were 36 (IQR, 21-62). Among the total contact days, specialty palliative care accounted for a median of 5 (IQR, 2-10) days and inpatient hospitalizations accounted for 17 (IQR, 9-29) days. The median percentage of contact days was 33.3%. For patients who did not receive systemic therapy, the median overall survival was 66 (IQR, 34-130) days and median (IQR) contact days were 28 (IQR, 17-44), with a median percentage of 42.4% contact days. Of these contact days, a median of 5 (IQR, 2-9) days were spent with specialty palliative care, and 6 (IQR, 5-11) days were spent with radiation oncology–related care (eTable 4 in Supplement 1). For patients who received systemic therapy, the median overall survival was 261 (IQR, 152-420) days and median contact days were 59 (IQR, 41-88), with a median percentage of 22.6% contact days. Of the patients receiving systemic treatment, 1115 (56.2%) received only cytotoxic chemotherapy (or trial/other therapy in <2% of these cases), 417 (21.0%) first- or subsequent-line immunotherapy, and 453 (22.8%) first- or subsequent-line targeted therapy. Among patients receiving 1 line of treatment, the median overall survival and contact days were cytotoxic chemotherapy (188 [IQR, 117-302] and 50 [IQR, 34-73]), immunotherapy (215 [IQR, 123-343] and 51 [IQR, 35-84]), and targeted therapy (269 [IQR, 113-418] and 52 [IQR, 33-69]) (eTable 4 in Supplement 1).

**Figure 1.**
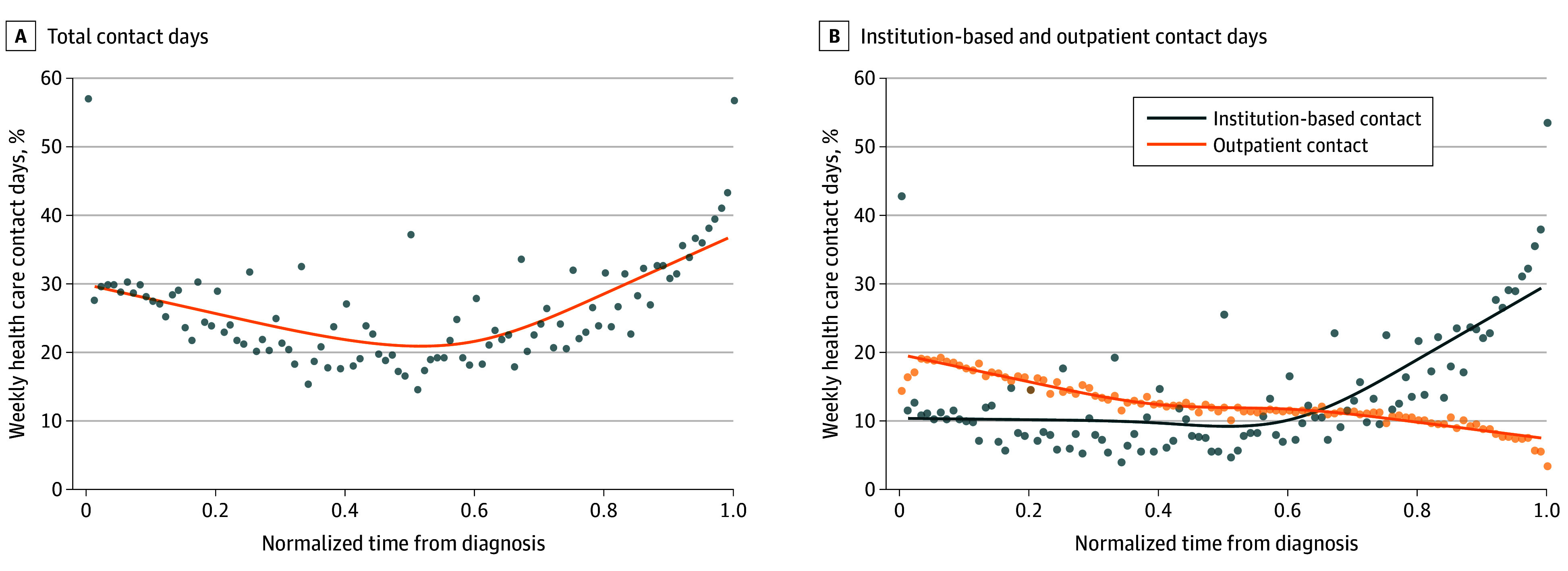
Overall Weekly Contact Days From Diagnosis to Death A, Total contact days. B, Institution-based and outpatient contact days. To ensure a fair comparison of contact day trajectories and facilitate visualization of contact day trajectories across patients with differential survival durations, irrespective of individual survival lengths, the time between non–small cell lung cancer diagnosis to death for each decedent was normalized. Cubic smoothing splines (lines) were fitted to estimate the trajectories of the time series observations. Average values of time series observations (dots) were also plotted.

**Figure 2.**
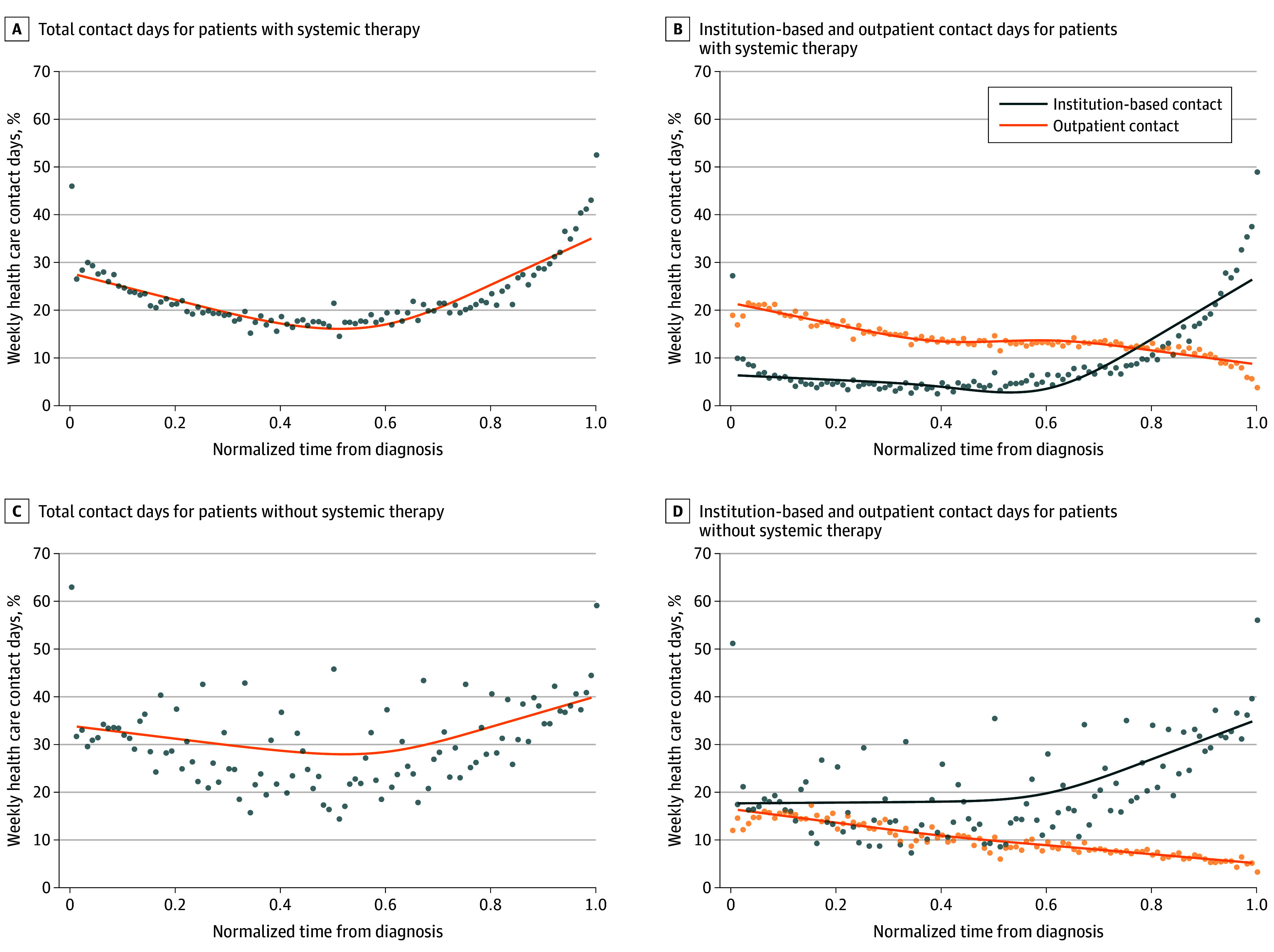
Weekly Contact Days, Stratified by Systemic Therapy Use A, Total contact days for patients with systemic therapy. B, Institution-based and outpatient contact days for patients with systemic therapy. C, Total contact days for patients without systemic therapy. D, Institution-based and outpatient contact days for patients without systemic therapy. To ensure a fair comparison of contact day trajectories and facilitate visualization of contact day trajectories across patients with differential survival durations, irrespective of individual survival lengths, the time between non–small cell lung cancer diagnosis to death for each decedent was normalized. Cubic smoothing splines (lines) were fitted to estimate the trajectories of the time series observations. Average values of time series observations (dots) were also plotted.

For the overall cohort, the percentage of weekly contact days followed a U-shaped normalized trajectory from diagnosis to death: an initial high start (29.4% contact days in the first month) followed by a trough phase (20.7%), which eventually led to a peak (36.5% contact days in the month before death) (Figure 1A). Outpatient contact days consistently declined from a high of 19.3% after diagnosis to 7.3% before death. Institution-based contact days were relatively stable at 10% until midway of the course, before a sharp increase to 29.2% before death. The overlay of outpatient and institution-based contact days is the basis of the U-shaped trajectory (Figure 1B). When comparing patients who received systemic therapy vs those who did not, the trajectory formed a more rounded and deeper U shape among patients receiving systemic therapy (Figure 2A, C). The trajectory for institution-based contact increased, while the one for the outpatient contact steadily decreased over time (Figure 2B, D). Trajectories for patients surviving 6 months or less and for those who did not receive systemic therapy were similar (eFigure 2 in Supplement 1). Trajectories were similar for patients based on time to initiation of systemic therapy (eFigure 3 in Supplement 1**)**. eTable 5 and eTable 6 in Supplement 1 present sociodemographic and clinical characteristics, and eTable 7 in Supplement 1 presents overall survival and health care contact days by survival and time to initiation of systemic therapy. Among patients receiving systemic therapy, the contact day rate was similar irrespective of the time to initiation of systemic therapy.

**Figure 3.**
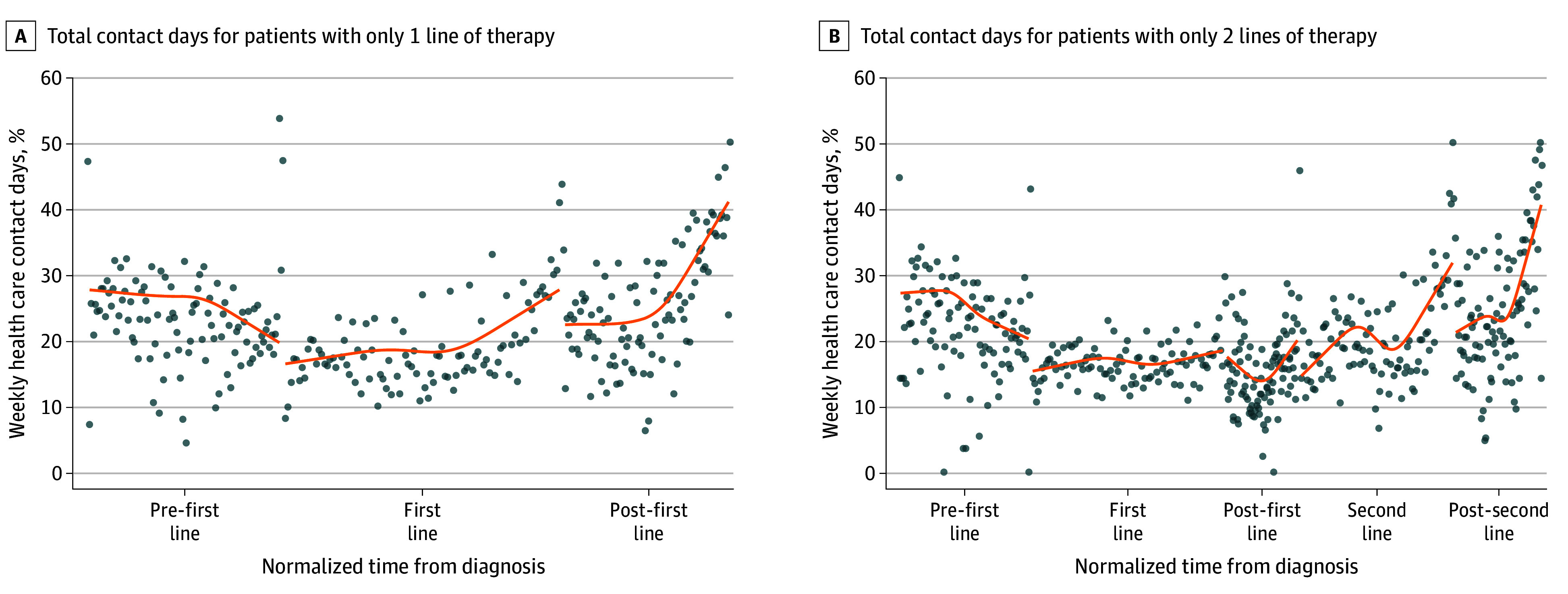
Weekly Contact Days, Stratified by Lines of Systemic Therapy A, Total contact days for patients with only 1 line of therapy. B, Total contact days for patients with only 2 lines of therapy. To ensure a fair comparison of contact day trajectories and facilitate visualization of contact day trajectories across patients with differential survival durations, irrespective of individual survival lengths, the time during each phase for each decedent was normalized. Cubic smoothing splines (lines) were fitted to estimate the trajectories of the time series observations. Average values of time series observations (dots) were also plotted.

**Figure 4.**
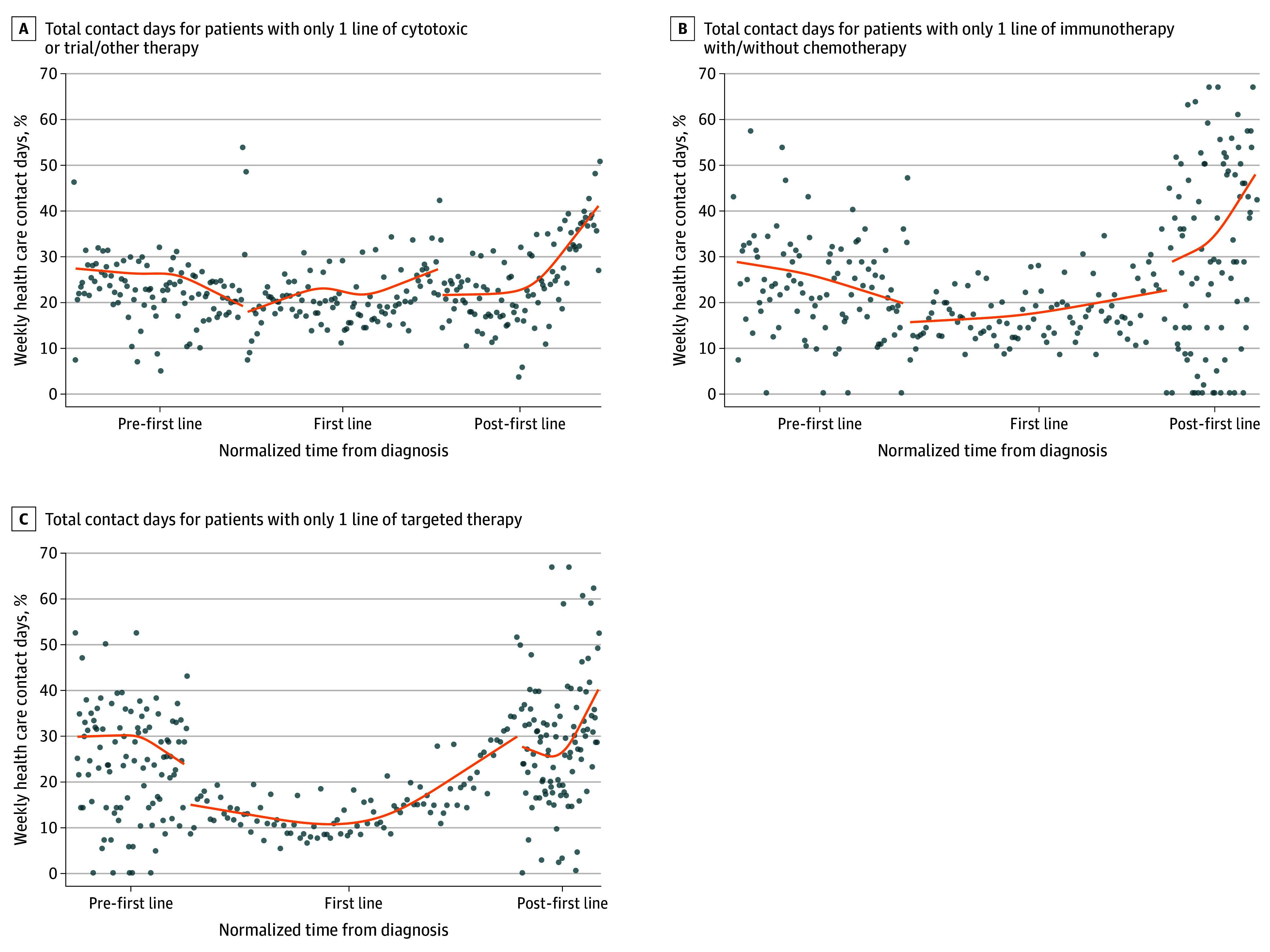
Weekly Contact Days, Stratified by Type of Systemic Therapy A, Total contact days for patients with only 1 line of cytotoxic or trial/other therapy. B, Total contact days for patients with only 1 line of immunotherapy with or without chemotherapy. C, Total contact days for patients with only 1 line of targeted therapy. To ensure a fair comparison of contact day trajectories and facilitate visualization of contact day trajectories across patients with differential survival durations, irrespective of individual survival lengths, the time during each phase for each decedent was normalized. Cubic smoothing splines (lines) were fitted to estimate the trajectories of the time series observations. Average values of time series observations (dots) were also plotted.

Among patients who received systemic therapy, an increase in contact days toward the end of the last line of therapy a patient received (first line in 1 line, and second line in 2 lines) was followed by a more acute rise in the posttherapy period leading into death (Figure 3). Among patients receiving 1 line of therapy, those who received targeted therapy (10.6% vs immunotherapy, 15.4% vs chemotherapy, 17.7%) experienced the deepest trough with fewest contact days (Figure 4).

For patients not receiving systemic therapy, urban residence and higher comorbidity burden were associated with greater contact days in the first month (eTable 8 in Supplement 1). Patients receiving targeted therapy compared with cytotoxic chemotherapy experienced more contact days in the first month but fewer contact days in the lowest contact month (eTable 9 in Supplement 1). Higher generalized physical symptom scores were associated with higher contact days in the first month, and higher mood-based symptom scores were associated with higher contact days in the lowest contact month (eTable 10 in Supplement 1).

## Discussion

In this population-based cohort study of 5785 lung cancer decedents, we found that patients had a median survival of 3.5 months and spent 1 in 3 of those days with health care contact outside the home. The percentage of weekly contact days overall followed a U-shaped normalized trajectory from diagnosis to death; outpatient days were the major source of the initial peak, while a steady increase in institution-based contact days midway onward accounted for the second peak. Patients receiving vs not receiving systemic cancer-directed treatment, and specifically, patients receiving targeted therapy vs cytotoxic chemotherapy experienced a deeper U shape trajectory. In addition to the treatment type, contact days also varied by sociodemographic characteristics, such as rural residence, and clinical factors, such as symptom severity. These results are a call for the oncology community to recognize time toxicity, provide better support for patients during periods of high burden, and benchmark appropriateness.

Despite a near universal U-shaped trajectory of contact days among subgroups, we observed important nuances in the rates, patterns, sources, and factors associated with contact days among patients who did not and those who did receive systemic treatment. We do not believe that delivering vs not delivering systemic therapy would alter the course of those patients. First, let us consider patients who did not receive systemic cancer-directed treatment—they accounted for two-thirds of the cohort, lived approximately 2 months, and spent 42.4% of their days alive receiving health care. Over 50% of this group who did not receive systemic therapy received palliative radiation, and in their roughly 2 months alive, they spent a median of 5 days with specialty palliative care and 6 days with radiation oncology–related care. The U trajectory was shallow: patients experienced persistently high contact days. Institution-based days steadily increased to account for almost all contact days at the end of life. These data indicate a sick population with high health care needs initially: these patients were older, had a higher comorbidity burden and symptom burden, and more commonly had squamous cell carcinoma (lower rates of driver mutations). In comparison with the 42.4% contact day rate reported herein, participants with advanced cancer in a clinical trial (including many from Ontario) receiving supportive care alone experienced a 6% contact days rate.^20^ This 42.4% (community practice setting) vs 6% (clinical trial) rate of contact days for supportive care alone may relate to differences in data sources, but importantly highlights the differences in patient populations and supportive care delivery efficiency.

Second, let us consider the 1985 patients who received systemic treatment. A total of 43.8% of them received immunotherapy or targeted therapy, reflecting uptake of novel treatments in the community setting.^16^ The median survival was 261 (IQR, 152-420) days and median contact days were 59 (IQR, 41-88), with a median percentage of 22.6% of contact days. This survival is similar to the 11.6-month survival in a contemporary French cohort and the 6-month median survival in US older adults with NSCLC and brain metastasis.^21,22^ The 29.4% contact day rate in the first month is in line with prior work that older adults with stage I NSCLC spend 1 in 3 days with health care contact postdiagnsosis.^23^ We observed that across subgroups, institution-based contact days increased an absolute of 20% from diagnosis to death. Whereas patients not receiving systemic therapy started at about 20%, patients receiving systemic therapy started at about 5%. Thus, high initial postdiagnosis rates of institution-based contact days may identify patients who are unlikely to receive systemic cancer treatment. The percentage of contact days was similar (approximately 20%) irrespective of whether systemic therapy was initiated within a month vs more than 3 months after diagnosis, although the longer overall survival (211 vs 339 days) in the latter group might reflect immortal time bias.

We specifically chose the study population and years based on the substantial increase in systemic treatment options for stage IV NSCLC in the mid-2010s.^2,16^ We conducted 3 analyses to critically characterize contact days by treatment-related factors. First, we compared the trajectories among patients who received 1 and 2 lines of treatment. The pre-first line and post-last line of treatment phases were similar in both groups, forming the initial descent and eventual sharp rise in contact days (the 2 arms of the U trajectory). The increase in contact days during the last line of treatment highlights how an increase in contact days during treatment may indicate that that line may be the last line that patients receive. Second, we noted significant differences in patterns during treatment by the type of treatment. While patients receiving cytotoxic chemotherapy and immunotherapy had relatively flatter and higher trajectories, patients receiving targeted therapy had a deeper trough. These patients may thus have a window with relatively lower time burdens, albeit only temporarily; eventually, these patients also experienced a rapid increase in contact days during treatment.^24^ Although patients receiving targeted therapy experienced the lowest trough of contact days, they experienced more contact days in the first month after diagnosis compared with patients receiving cytotoxic chemotherapy. This might relate to the more intensive workup for patients suspected of having driver alterations^25^ and their often more serious clinical presentation (multiple brain metastases).

Third, we additionally found that symptom severity was associated with contact days among patients receiving systemic therapy. Specifically, the association of mood-based symptom severity with contact days during the month in which patients experienced the fewest contact days emphasizes the need for psychosocial support even when patients are doing well and needing the least amount of health care than in other phases.^26^

### Limitations

This study has limitations. First, we lacked laboratory data from 1 of the 14 health regions, accounting for 14.3% of the study cohort, but were still able to capture most contact days using alternative data sources. We included only 8 common laboratory tests; thus, the current contact days could be an underrepresentation. Despite this, we overcame a major limitation of prior single-center electronic health record–based studies: that those studies could not account for contact days outside that center.^15^ Second, the cohort studied represents a community practice population in a single Canadian province over 2014-2019, and contact day patterns in the US may differ due to different practice and payment patterns.^27^ By including only decedents and limiting follow-up to 2 years, we might have biased the sample to those with shorter survival. However, this censoring at 2 years affected only 12.7% of the patients. The population included few patients enrolled in clinical trials. We did not delineate if patients primarily received cancer care in community and academic settings. While the study period was recent, the past 5 years have seen further developments in NSCLC therapeutics. Third, this study did not seek to gauge the quality of contact days, ie, whether a contact day was necessary or aligning with patients’ goals or preferences. How contact days also represent access to oftentimes necessary care has been previously highlighted.^28^ Fourth, one-third of patients received systemic therapy, which, while low, is in line with prior studies highlighting barriers to community practice setting eligibility for and receipt of cancer-directed treatments.^27,29^ Fifth, we were unable to identify whether institution-based contact days included hospice care. Our ongoing qualitative work seeks to determine whether and how to include home-based care (eg, telemedicine appointments, home infusions) into the contact day measure.

## Conclusions

In this cohort study of patients with stage IV NSCLC diagnosed in the mid-2010s onward, a period during which several immunotherapy and targeted therapy options were available, we found that patients had a median survival of 3.5 months and spent 1 in 3 days with health care contact outside the home. Contact days followed a U-shaped normalized trajectory over time. Additional novel findings included that the immediate postdiagnosis rate of institution-based contact days (high vs low) may identify patients at risk of never receiving cancer treatment and short survival, and an increase in contact days during systemic treatment may indicate that may be the last line of treatment. We observed that contact days varied by sociodemographic factors, such as rural residence, and clinical factors, such as comorbidity and symptom burden. These data suggest the need to recognize patient time toxicity, improve care delivery efficiency, and provide better support for patients during periods of high burden, while providing additional research and improvement opportunities, such as proactive interventions before upward inflections in contact days.

## Notes

Supplement 1. **eTable 1.** *International Classification of Disease O-3* (*ICD-O-3*) Morphology and Topography Codes for Non–Small Cell Lung Cancer (NSCLC) Diagnoses **eTable 2.** Classification of Systemic Therapy Administered for Patients Diagnosed With Stage IV Non–Small Cell Lung Cancer (NSCLC) From January 1, 2014, to December 31, 2017, in Ontario, Canada **eTable 3.** Descriptions of the ICES Health Administrative Databases, Outcomes and Covariates **eTable 4.** Overall Survival and Specific Contact Days for Patients Diagnosed With Stage IV Non–Small Cell Lung Cancer (NSCLC) From January 1, 2014, to December 31, 2017, in Ontario, Canada, Stratified by Type of Systemic Therapy **eTable 5.** Sociodemographic and Clinical Characteristics for Patients Diagnosed With Stage IV Non–Small Cell Lung Cancer (NSCLC) From January 1, 2014, to December 31, 2017, in Ontario, Canada, Stratified by Overall Survival **eTable 6.** Sociodemographic and Clinical Characteristics, for Patients Diagnosed With Stage IV Non–Small Cell Lung Cancer (NSCLC) From January 1, 2014, to December 31, 2017, and Receiving Systemic Therapy from January 1, 2014, to December 31, 2019, in Ontario, Canada, Stratified by Time to Initiation of Systemic Therapy From Cancer Diagnosis **eTable 7.** Overall Survival and Contact Days for Patients Diagnosed With Stage IV Non–Small Cell Lung Cancer (NSCLC) From January 1, 2014, to December 31, 2017, in Ontario, Canada, Stratified by Overall Survival and Time From Diagnosis to Systemic Therapy Initiation **eTable 8.** Multivariable Analyses for Healthcare Contact Days in Specific Months for Patients Diagnosed With Stage IV Non–Small Cell Lung Cancer (NSCLC) From January 1, 2014, to December 31, 2017, and Not Receiving Systemic Therapy From January 1, 2014, to December 31, 2019, in Ontario, Canada **eTable 9.** Multivariable Analyses for Healthcare Contact Days in Specific Months for Patients Diagnosed With Stage IV Non–Small Cell Lung Cancer (NSCLC) From January 1, 2014, to December 31, 2017, and Receiving Systemic Therapy From January 1, 2014, to December 31, 2019, in Ontario, Canada **eTable 10.** Multivariable Analyses for Healthcare Contact Days in Specific Months for Patients Diagnosed With Stage IV Non–Small Cell Lung Cancer (NSCLC) From January 1, 2014, to December 31, 2017, Receiving Systemic Therapy From January 1, 2014, to December 31, 2019, and Completing ESAS Assessments in Ontario, Canada **eFigure 1.** Cohort Creation of Patients Diagnosed With Stage IV Non–Small Cell Lung Cancer (NSCLC) From January 1, 2014, to December 31, 2017, in Ontario, Canada **eFigure 2.** Weekly Contact Days, Stratified by Survival, for Patients Diagnosed With Stage IV Non–Small Cell Lung Cancer (NSCLC) From January 1, 2014, to December 31, 2017, in Ontario, Canada **eFigure 3.** Weekly Contact Days, Stratified by Time to Initiation of Systemic Therapy, for Patients Diagnosed With Stage IV Non–Small Cell Lung Cancer (NSCLC) From January 1, 2014, to December 31, 2017, in Ontario, Canada

Supplement 2.
Data Sharing Statement
