# Supplementary material for: Trajectories of Health Care Contact Days for Patients With Stage IV Non–Small Cell Lung Cancer
Source: JAMA Netw Open. 2024 Apr 8;7(4):e244278. doi: 10.1001/jamanetworkopen.2024.4278 (PMC11002696; doi:10.1001/jamanetworkopen.2024.4278)
Supplement: Supplement 1. — eTable 1. International Classification of Disease O-3 (ICD-O-3) Morphology and Topography Codes for Non–Small Cell Lung Cancer (NSCLC) Diagnoses eTable 2. Classification of Systemic Therapy Administered for Patients Diagnosed With Stage IV Non–Small Cell Lung Cancer (NSCLC) From January 1, 2014, to December 31, 2017, in Ontario, Canada eTable 3. Descriptions of the ICES Health Administrative Databases, Outcomes and Covariates eTable 4. Overall Survival and Specific Contact Days for Patients Diagnosed With Stage IV Non–Small Cell Lung Cancer (NSCLC) From January 1, 2014, to December 31, 2017, in Ontario, Canada, Stratified by Type of Systemic Therapy eTable 5. Sociodemographic and Clinical Characteristics for Patients Diagnosed With Stage IV Non–Small Cell Lung Cancer (NSCLC) From January 1, 2014, to December 31, 2017, in Ontario, Canada, Stratified by Overall Survival eTable 6. Sociodemographic and Clinical Characteristics, for Patients Diagnosed With Stage IV Non–Small Cell Lung Cancer (NSCLC) From January 1, 2014, to December 31, 2017, and Receiving Systemic Therapy from January 1, 2014, to December 31, 2019, in Ontario, Canada, Stratified by Time to Initiation of Systemic Therapy From Cancer Diagnosis eTable 7. Overall Survival and Contact Days for Patients Diagnosed With Stage IV Non–Small Cell Lung Cancer (NSCLC) From January 1, 2014, to December 31, 2017, in Ontario, Canada, Stratified by Overall Survival and Time From Diagnosis to Systemic Therapy Initiation eTable 8. Multivariable Analyses for Healthcare Contact Days in Specific Months for Patients Diagnosed With Stage IV Non–Small Cell Lung Cancer (NSCLC) From January 1, 2014, to December 31, 2017, and Not Receiving Systemic Therapy From January 1, 2014, to December 31, 2019, in Ontario, Canada eTable 9. Multivariable Analyses for Healthcare Contact Days in Specific Months for Patients Diagnosed With Stage IV Non–Small Cell Lung Cancer (NSCLC) From January 1, 2014, to December 31, 2017, and Receiving System [file jamanetwopen-e244278-s001.pdf]

Supplementary Online Content

Gupta A, Nguyen P, Kain D, et al. Trajectories of health care contact days for patients with stage IV non–small cell lung cancer. *JAMA Netw Open*. 2024;7(4):e244278. doi:10.1001/jamanetworkopen.2024.4278

eTable 1. *International Classification of Disease O-3* (*ICD-O-3*) Morphology and Topography Codes for Non–Small Cell Lung Cancer (NSCLC) Diagnoses

eTable 2. Classification of Systemic Therapy Administered for Patients Diagnosed With Stage IV Non–Small Cell Lung Cancer (NSCLC) From January 1, 2014, to December 31, 2017, in Ontario, Canada

eTable 3. Descriptions of the ICES Health Administrative Databases, Outcomes and Covariates

eTable 4. Overall Survival and Specific Contact Days for Patients Diagnosed With Stage IV Non–Small Cell Lung Cancer (NSCLC) From January 1, 2014, to December 31, 2017, in Ontario, Canada, Stratified by Type of Systemic Therapy

eTable 5. Sociodemographic and Clinical Characteristics for Patients Diagnosed With Stage IV Non-Small Cell Lung Cancer (NSCLC) From January 1, 2014, to December 31, 2017, in Ontario, Canada, stratified by overall survival

eTable 6. Sociodemographic and Clinical Characteristics, for Patients Diagnosed With Stage IV Non–Small Cell Lung Cancer (NSCLC) From January 1, 2014, to December 31, 2017, and Receiving Systemic Therapy from January 1, 2014, to December 31, 2019, in Ontario, Canada, Stratified by Time to Initiation of Systemic Therapy From Cancer Diagnosis

eTable 7. Overall Survival and Contact Days for Patients Diagnosed With Stage IV Non–Small Cell Lung Cancer (NSCLC) From January 1, 2014, to December 31, 2017, in Ontario, Canada, Stratified by Overall Survival and Time From Diagnosis to Systemic Therapy Initiation

eTable 8. Multivariable Analyses for Healthcare Contact Days in Specific Months for Patients Diagnosed With Stage IV Non–Small Cell Lung Cancer (NSCLC) From January 1, 2014, to December 31, 2017, and Not Receiving Systemic Therapy From January 1, 2014, to December 31, 2019, in Ontario, Canada

eTable 9. Multivariable Analyses for Healthcare Contact Days in Specific Months for Patients Diagnosed With Stage IV Non–Small Cell Lung Cancer (NSCLC) From January 1, 2014, to December 31, 2017, and Receiving Systemic Therapy From January 1, 2014, to December 31, 2019, in Ontario, Canada

eTable 10. Multivariable Analyses for Healthcare Contact Days in Specific Months for Patients Diagnosed With Stage IV Non–Small Cell Lung Cancer (NSCLC) From January 1, 2014, to December 31, 2017, Receiving Systemic Therapy From January 1, 2014, to December 31, 2019, and Completing ESAS Assessments in Ontario, Canada

eFigure 1. Cohort Creation of Patients Diagnosed With Stage IV Non–Small Cell Lung Cancer (NSCLC) From January 1, 2014, to December 31, 2017, in Ontario, Canada

eFigure 2. Weekly Contact Days, Stratified by Survival, for Patients Diagnosed With Stage IV Non–Small Cell Lung Cancer (NSCLC) From January 1, 2014, to December 31, 2017, in Ontario, Canada

eFigure 3. Weekly Contact Days, Stratified by Time to Initiation of Systemic Therapy, for Patients Diagnosed With Stage IV Non–Small Cell Lung Cancer (NSCLC) From January 1, 2014, to December 31, 2017, in Ontario, Canada

This supplementary material has been provided by the authors to give readers additional information about their work.

© 2024 Gupta A et al. *JAMA Network Open.*

eTable 1: *International Classification of Disease O-3* (*ICD-O-3*) morphology and topography codes for non-small cell lung cancer (NSCLC) diagnoses

ICD-O-3 Code	ICD-O-3 Description
Morphology	
8000/3	Neoplasm, malignant
8001/3	Tumor cells, malignant
8004/3	Malignant tumor, spindle cell type
8010/3	Carcinoma, NOS
8012/3	Large cell carcinoma, NOS
8020/3	Carcinoma, undifferentiated, NOS
8021/3	Carcinoma, anaplastic, NOS
8022/3	Pleomorphic carcinoma
8030/3	Giant cell and spindle cell carcinoma
8031/3	Giant cell carcinoma
8032/3	Spindle cell carcinoma, NOS
8034/3	Polygonal cell carcinoma
8050/3	Papillary carcinoma, NOS
8051/3	Verrucous carcinoma, NOS
8052/3	Papillary squamous cell carcinoma
8070/3	Squamous cell carcinoma, NOS
8070/6	Squamous cell carcinoma, metastatic, NOS
8071/3	Squamous cell carcinoma, keratinizing, NOS
8072/3	Squamous cell carcinoma, large cell, nonkeratinizing, NOS
8073/3	Squamous cell carcinoma, small cell, nonkeratinizing
8074/3	Squamous cell carcinoma, spindle cell
8075/3	Squamous cell carcinoma, adenoid
8076/3	Squamous cell carcinoma, microinvasive
8082/3	Lymphoepithelial carcinoma
8094/3	Basosquamous carcinoma
8120/3	Transitional cell carcinoma, NOS
8130/3	Papillary transitional cell carcinoma
8140/3	Adenocarcinoma, NOS
8140/6	Adenocarcinoma, metastatic, NOS
8141/3	Scirrhous adenocarcinoma
8143/3	Superficial spreading adenocarcinoma
8144/3	Adenocarcinoma, intestinal type
8145/3	Carcinoma, diffuse type
8190/3	Trabecular adenocarcinoma
8200/3	Adenoid cystic carcinoma
8201/3	Cribriform carcinoma, NOS
8210/3	Adenocarcinoma in adenomatous polyp
8211/3	Tubular adenocarcinoma
8230/3	Solid carcinoma, NOS
8231/3	Carcinoma simplex
8250/3	Bronchiolo-alveolar adenocarcinoma, NOS
8251/3	Alveolar adenocarcinoma
8260/3	Papillary adenocarcinoma, NOS
8261/3	Adenocarcinoma in villous adenoma
8263/3	Adenocarcinoma in tubulovillous adenoma
8290/3	Oxyphilic adenocarcinoma
8310/3	Clear cell adenocarcinoma, NOS
8323/3	Mixed cell adenocarcinoma

© 2024 Gupta A et al. *JAMA Network Open.*

8330/3	Follicular adenocarcinoma, NOS
8340/3	Papillary carcinoma, follicular variant
8380/3	Endometrioid carcinoma, NOS
8401/3	Apocrine adenocarcinoma
8410/3	Sebaceous adenocarcinoma
8420/3	Ceruminous adenocarcinoma
8430/3	Mucoepidermoid carcinoma
8440/3	Cystadenocarcinoma, NOS
8441/3	Serous cystadenocarcinoma, NOS
8442/3	Serous cystadenoma, borderline malignancy
8462/3	Papillary serous cystadenoma, borderline malignancy
8470/3	Mucinous cystadenocarcinoma, NOS
8472/3	Mucinous cystadenoma, borderline malignancy
8480/3	Mucinous adenocarcinoma
8481/3	Mucin-producing adenocarcinoma
8490/3	Signet ring cell carcinoma
8490/6	Metastatic signet ring cell carcinoma
8500/3	Infiltrating duct carcinoma
8510/3	Medullary carcinoma, NOS
8550/3	Acinar cell carcinoma
8560/3	Adenosquamous carcinoma
8562/3	Epithelial-myoepithelial carcinoma
8570/3	Adenocarcinoma with squamous metaplasia
8572/3	Adenocarcinoma with spindle cell metaplasia
8802/3	Giant cell sarcoma
8980/3	Carcinosarcoma, NOS
Topography	
C34.0	Main bronchus
C34.1	Upper lobe, lung
C34.2	Middle lobe, lung
C34.3	Lower lobe, lung
C34.8	Overlapping lesion of lung
C34.9	Lung, NOS

Acronyms:
NOS, not otherwise specified

© 2024 Gupta A et al. *JAMA Network Open.*

eTable 2: Classification of systemic therapy administered for patients diagnosed with stage IV non-small cell lung cancer (NSCLC) from January 1, 2014, to December 31, 2017, in Ontario, Canada

Regimen Class	Regimen Description
Cytotoxic therapy	
Single-agent	Abraxane[a], Carboplatin, Cisplatin, Cyclophosphamide[a], Docetaxel, Etoposide, Gemcitabine, Paclitaxel, Pemetrexed, Vinorelbine
Multi-agent	Carboplatin + Abraxane[a]<br>Carboplatin + Docetaxel<br>Carboplatin + Etoposide<br>Carboplatin + Gemcitabine<br>Carboplatin + Paclitaxel<br>Carboplatin + Pemetrexed<br>Carboplatin + Vinblastine<br>Carboplatin + Vinorelbine<br>Carboplatin + Cisplatin + Etoposide[a]<br>Carboplatin + Cyclophosphamide + Pemetrexed[a]<br>Cisplatin + Etoposide<br>Cisplatin + Gemcitabine<br>Cisplatin + Pemetrexed<br>Cisplatin + Vinblastine[a]<br>Cisplatin + Vinorelbine<br>Cyclophosphamide + Doxorubicin + Vincristine[a]
Trial, or other therapy	
Single-agent	Bevacizumab[a], Binimetinib[a], Ceralasertib[a], CFI-402257[a], MG1MA3[a], Nintedanib[a], Onalespib[a]
Multi-agent	Carboplatin + Paclitaxel + Bevacizumab[a]<br>Carboplatin + Paclitaxel + Selumetinib[a]<br>Carboplatin + Paclitaxel + Veliparib[a]<br>Carboplatin + Pemetrexed + Bevacizumab[a]<br>Carboplatin + Pemetrexed + Selumetinib[a]<br>Cisplatin + Pemetrexed + Selumetinib
Immunotherapy	
Single-agent	Atezolizumab[a], Durvalumab, Ipilimumab, Nivolumab, Pembrolizumab
Multi-agent	Durvalumab + Tremelimumab<br>Ipilimumab + Nivolumab[a]
Immunotherapy with chemotherapy	
Multi-agent	Durvalumab + Carboplatin + Gemcitabine[a]<br>Durvalumab + Cisplatin + Pemetrexed[a]<br>Durvalumab + Pemetrexed<br>Durvalumab + Tremelimumab + Carboplatin + Abraxane[a]<br>Durvalumab + Tremelimumab + Carboplatin + Gemcitabine[a]<br>Durvalumab + Tremelimumab + Carboplatin + Pemetrexed<br>Durvalumab + Tremelimumab + Cisplatin + Gemcitabine[a]<br>Durvalumab + Tremelimumab + Cisplatin + Pemetrexed<br>Durvalumab + Tremelimumab + Pemetrexed<br>Nivolumab + Cisplatin + Pemetrexed[a]<br>Nivolumab + Pemetrexed[a]
Targeted therapy	
Single-agent	Afatinib, Alectinib[a], Ceritinib, Crizotinib, Erlotinib, Gefitinib, Lorlatinib[a], Osimertinib
Multi-agent	Afatinib + Erlotinib[a]<br>Gefitinib + Erlotinib[a]<br>Gefitinib + Osimertinib[a]

© 2024 Gupta A et al. *JAMA Network Open.*

Notes:
a. Single-agent or multi-agent systemic therapy was administered for ≤5 patients.

© 2024 Gupta A et al. *JAMA Network Open.*

eTable 3: Descriptions of the ICES health administrative databases, outcomes and covariates

Database	Source	Description
Registered Persons Database (RPDB)	MOH	It contains basic demographic information (e.g., age, sex, date of birth, and date of death for deceased individuals) for those issued an Ontario health insurance number.
Ontario Cancer Registry (OCR)	OH	It contains information for all Ontario residents who have been diagnosed with cancer or who have died of cancer.
Ontario Health Insurance Plan (OHIP) Database	MOH	It contains claims and associated diagnoses paid for inpatient and outpatient services (e.g., physicians, and laboratories) provided to Ontario residents eligible for the publicly funded health insurance system.
Discharge Abstract Database (DAD)	CIHI, MOH	It contains administrative (e.g., institution, admission category, length of stay, and disposition), clinical (e.g., diagnoses, and procedures/interventions), and demographic (e.g., age, gender, and location of residence) information for all inpatient admissions to acute care hospitals.
Ontario Mental Health Reporting System (OMHRS)	CIHI, MOH	It contains information on patients in adult designated inpatient mental health beds.
Same Day Surgery (SDS) Database	CIHI, MOH	It contains administrative (e.g., institution, and disposition), clinical (e.g., diagnoses, and procedures/interventions), and demographic (e.g., age, gender, and location of residence) information for all patient visits made to day surgery institutions.
National Ambulatory Care Reporting System (NACRS)	CIHI, MOH	It contains administrative (e.g., institution, and disposition), clinical (e.g., diagnoses, and procedures/interventions), and demographic (e.g., age, gender, and location of residence) information for all patient visits made to hospital- and community-based ambulatory care centers (e.g., emergency departments, hemodialysis units, and cancer care clinics).
Continuing Care Reporting System (CCRS)	CIHI, MOH	It contains clinical (e.g., RAI-MDS 2.0 assessment) and demographic (e.g., primary language spoken, and marital status) information for Ontario residents receiving facility-based continuing care (also known as extended, auxiliary, or complex chronic care) in hospitals or long-term care homes.
National Rehabilitation Reporting System (NRS)	MOH	It contains information on patients in adult designated inpatient rehabilitation facilities and programs.
Ontario Laboratory Information System (OLIS)	MOH	It contains information from an Ontario-wide electronic repository of lab test orders and results from hospitals, community labs and public health labs.
Home Care Database (HCD)	HSSO	It contains information on clients, intake, assessment, admission, diagnostic and surgical procedure, and service delivery who received home care visits.
Cancer Activity Level Reporting (ALR)	OH	It contains information for patient-level activity within the cancer system focused on radiation and systemic therapy services and outpatient oncology clinic visits.
New Drug Funding Program (NDFP)	OH	It contains information for publicly funded high-quality intravenous cancer drugs.
Ontario Drug Benefit (ODB) Program	MOH	It contains prescription medication claims for those covered under the provincial drug program (e.g., residents aged ≥65 years, and receiving social assistance).

© 2024 Gupta A et al. *JAMA Network Open.*

Drugs List (DIN)	IQIVIA Solutions Canada Inc, MOH	It contains drug information on product names, subclass, strength, route of administration, and first and last dispensing dates from the ODB data.
Symptom Management Database (ESAS)	OH	It contains information from a web-based symptom screening tool for healthcare providers to monitor patient-level symptoms and performance status.
Canadian Community Health Survey (CCHS)	Statistics Canada, MOH	It contains survey-collected information for health status, healthcare utilization and health determinants of randomly sampled Ontario residents.
Ontario Asthma (ASTHMA) Database	CIHI, MOH	It identifies Ontario residents with asthma based on an ICES-derived validated algorithm using the OHIP, DAD and SDS data.
Ontario Chronic Obstructive Pulmonary Disease (COPD) Database	CIHI, MOH	It identifies Ontario residents with chronic obstructive pulmonary disease (COPD) based on an ICES-derived validated algorithm using the OHIP, DAD and SDS data.
Ontario Hypertension (HYPER) Database	CIHI, MOH	It identifies Ontario residents with hypertension based on an ICES-derived validated algorithm using the OHIP, DAD and SDS data.
Ontario Congestive Heart Failure (CHF) Database	CIHI, MOH	It identifies Ontario residents with congestive heart failure (CHF) based on an ICES-derived validated algorithm using the OHIP, DAD and SDS data.
Ontario Dementia (DEMENTIA) Database	CIHI, MOH	It identifies Ontario adults aged ≥40 years with dementia based on an ICES-derived validated algorithm using the OHIP, DAD, SDS and ODB data.

Acronyms:
MOH, Ministry of Health; OH, Ontario Health; CIHI, Canadian Institute for Health Information; RAI-MDS, Resident Assessment Instrument Minimum Data Set; HSSO, Health Shared Services Ontario

Descriptions were drawn from the ICES Intranet with adaption from the following references:

1. ICES. ICES data dictionary. https://datadictionary.ices.on.ca. Accessed January 10, 2023. 2023. 2. ICES Privacy & Legal Office. ICES Report to the Information and Privacy Commissioner of Ontario: Three-Year Review as a Prescribed Entity under PHIPA, 2020.

Outcomes

Healthcare contact days were identified with any health administrative records for inpatient acute or rehabilitation hospitalizations (DAD, OMHRS, NRS); emergency department visits (NACRS); outpatient surgeries (SDS); cancer clinic visits (NACRS); dialysis clinic visits (NACRS); long-term or complex continuing care (CCRS); outpatient contact (e.g., family physician, imaging, injections/infusions, and radiotherapy assessment and treatments) (OHIP); and blood lab test visits (OLIS) from the selected ICES databases. Healthcare contact was counted once per day regardless of the duration, location or reason of contact.

Covariates

Socioeconomic status was based on community-specific or neighborhood household income quintiles. Rurality of residence was classified using the 2008 Rurality Index for Ontario (RIO), with higher scores representing a greater degree of rurality: Rural area (RIO score ≥40), suburban area (10≤ RIO score <40), and urban centers (RIO score <10) (1). Smoking status

© 2024 Gupta A et al. *JAMA Network Open.*

was based on survey respondents in the 2015-2017 Canadian Community Health Survey (CCHS) data; specifically, area-level age-sex standardized rates were computed with the 2016 Ontario census division (CD) geography and population to represent the areal smoking status (i.e., higher than average, typical average or lower than average) for their place of residence. Comorbidities were measured using the Elixhauser comorbidity index derived from hospital records from the DAD and SDS databases with a 5-year lookback from their NSCLC diagnosis (2). Chronic conditions (e.g., asthma, hypertension, and dementia) were based on the ICES-derived databases.

Systemic therapy was described with specific anticancer medications from the ALR, NDFP and ODB databases, while radiotherapy was defined with specific treatment activities from the ALR database. Metastasis surgeries were identified from cancer-related surgical interventions from the DAD database related to brain resection or spinal cord compression. Palliative systemic therapy, radiotherapy and metastasis surgery treatment were measured from NSCLC diagnosis to death.

References

1. Kralj B. Ontario Medical Association. Measuring Rurality - RIO 2008_BASIC: Methodology and Results. Toronto ON: Ontario Medical Association Economics Department 2009.
2. Moore BJ, White S, Washington R, et al. Identifying increased risk of readmission and in-hospital mortality using hospital administrative data. Medical care 2017; 55: 698-705.

© 2024 Gupta A et al. *JAMA Network Open.*

eTable 4: Overall survival and specific contact days for patients diagnosed with stage IV non-small cell lung cancer (NSCLC) from January 1, 2014, to December 31, 2017, in Ontario, Canada, stratified by type of systemic therapy

	Total (N=5,785)	Systemic Therapy (N=1,985)	No Systemic Therapy (N=3,800)	No Systemic Therapy (N=3,800)		One Line of Systemic Therapy (N=1,214)		
**Median (IQR), days**				**Radiation Therapy (N=2,112)**	**No Radiation Therapy (N=1,688)**	**Cytotoxic, or Trial/Other (N=921)**	**Immunotherapy (N=70)**	**Targeted Therapy (N=223)**
Overall survival, days	108 (49, 246)	261 (152, 420)	66 (34, 130)	88 (52, 158)	43 (21, 84)	188 (117, 302)	215 (123, 343)	269 (113, 418)
Total contact days	36 (21, 62)	59 (41, 88)	28 (17, 44)	33 (23, 51)	20 (11, 33)	50 (34, 73)	51 (35, 84)	52 (33, 69)
Radiation oncology related contact days	7 (5, 12)	11 (6, 15)	6 (5, 11)	6 (5, 11)	1 (1, 2)	10 (6, 14)	11 (6, 13)	10 (6, 12.5)
Specialty palliative care contact days	5 (2, 10)	6 (3, 12)	5 (2, 9)	5 (2, 10)	4 (1, 7)	5 (2.5, 11)	7 (3, 11)	6 (3, 11.5)

Acronyms:
IQR, interquartile range

© 2024 Gupta A et al. *JAMA Network Open.*

eTable 5: Sociodemographic and clinical characteristics for patients diagnosed with stage IV non-small cell lung cancer (NSCLC) from January 1, 2014, to December 31, 2017, in Ontario, Canada, stratified by overall survival

Patient Characteristics	Total[a]	Overall Survival[a]	
		≤6 Months	>6 Months
	N=5,785	N=3,844	N=1,941
**Sociodemographic Characteristics**			
Age			
Median (IQR)	70 (62, 77)	70 (62, 78)	68 (61, 76)
20-59	1,011 (17.5%)	616 (16.0%)	395 (20.4%)
60-69	1,845 (31.9%)	1,174 (30.5%)	671 (34.6%)
70-79	1,870 (32.3%)	1,273 (33.1%)	597 (30.8%)
≥80	1,059 (18.3%)	781 (20.3%)	278 (14.3%)
Sex			
Female	2,677 (46.3%)	1,721 (44.8%)	956 (49.3%)
Male	3,108 (53.7%)	2,123 (55.2%)	985 (50.7%)
Income quintile			
1 (Lowest)	1,502 (26.0%)	1,044 (27.2%)	458 (23.6%)
2	1,331 (23.0%)	874 (22.7%)	457 (23.5%)
3	1,061 (18.3%)	713 (18.5%)	348 (17.9%)
4	993 (17.2%)	646 (16.8%)	347 (17.9%)
5 (Highest)	877 (15.2%)	552 (14.4%)	325 (16.7%)
Urban/rural residence			
Urban (RIO<10)	3,643 (63.0%)	2,439 (63.4%)	1,204 (62.0%)
Suburban (10≤RIO<40)	1,458 (25.2%)	965 (25.1%)	493 (25.4%)
Rural (RIO≥40)	595 (10.3%)	385 (10.0%)	210 (10.8%)
Chronic conditions[b]			
Asthma	771 (13.3%)	500 (13.0%)	271 (14.0%)
COPD	2,433 (42.1%)	1,642 (42.7%)	791 (40.8%)
Hypertension	3,438 (59.4%)	2,331 (60.6%)	1,107 (57.0%)
CHF	554 (9.6%)	392 (10.2%)	162 (8.3%)
Dementia	179 (3.1%)	134 (3.5%)	45 (2.3%)
CKD	554 (9.6%)	416 (10.8%)	138 (7.1%)
**Clinical Characteristics**			
Histology/morphology			
Neoplasms, NOS	1,264 (21.8%)	913 (23.8%)	351 (18.1%)
Squamous cell neoplasms	1,003 (17.3%)	675 (17.6%)	328 (16.9%)
Adenomas or adeno-carcinomas	3,341 (57.8%)	2,147 (55.9%)	1,194 (61.5%)
Other	177 (3.1%)	109 (2.8%)	68 (3.5%)
ESAS assessment[c]			
Number of assessments			
N	3,451 (59.7%)	1,869 (48.6%)	1,582 (81.5%)
Median (IQR)	3 (1, 5)	2 (1, 4)	3 (2, 5)
Localized physical symptom score			
Median (IQR)	7 (4, 8)	7 (5, 9)	6 (3, 8)
Generalized physical symptom score			
Median (IQR)	8 (5, 9)	8 (7, 9)	7 (5, 8)
Mood-based symptom score			
Median (IQR)	5 (2, 7)	5 (2, 8)	4 (2, 7)

© 2024 Gupta A et al. *JAMA Network Open.*

Nearest cancer center from place of residence[d]			
Estimated shortest driving distance (in kilometers)			
Median (IQR)	22 (8, 56)	22 (8, 56)	22 (9, 57)
Estimated shortest driving duration (in minutes)			
Median (IQR)	25 (14, 47)	25 (14, 47)	25 (15, 47)

Acronyms:
IQR, interquartile range; RIO, Rurality Index for Ontario; COPD, chronic obstructive pulmonary disease; CHF, congestive heart failure; CKD, chronic kidney disease; NOS, not otherwise specified; ESAS, Edmonton Symptom Assessment System

Notes:

a. Column percentages may not sum to 100% due to missing data.
b. Chronic kidney disease (CKD) was measured with an average estimated glomerular filtration rate (eGFR) of <60 mL/min/1.73 m^2 from multiple laboratory tests within a 1-year lookback period from NSCLC diagnosis.
c. ESAS assessments were measured within a 3-month lookback and lookforward periods from NSCLC diagnosis; ESAS symptoms were categorized as localized physical (pain, nausea and shortness of breath), generalized physical (tiredness, drowsiness, lack of appetite and wellbeing) and mood-based symptom clusters (anxiety and depression); maximum intensity scores from any ESAS symptom within the cluster were used when multiple assessments were reported.
d. Driving distance and duration were measured with the shortest distance generated from the Open Source Routing Machine (OSRM) API with OpenStreetMap data between the postal code of residence and the geographic location of the regional cancer center.

© 2024 Gupta A et al. *JAMA Network Open.*

eTable 6: Sociodemographic and clinical characteristics, for patients diagnosed with stage IV non-small cell lung cancer (NSCLC) from January 1, 2014, to December 31, 2017, and receiving systemic therapy from January 1, 2014, to December 31, 2019, in Ontario, Canada, stratified by time to initiation of systemic therapy from cancer diagnosis

Patient Characteristics	Total[a]	Time from NSCLC Diagnosis to Systemic Therapy Initiation[a]			
		≤1 Month	>1-to-2 Months	>2-to-3 Months	>3 Months
	N=1,985	N=289	N=871	N=464	N=361
**Sociodemographic Characteristics**					
Age					
Median (IQR)	66 (60, 72)	65 (59, 71)	66 (60, 73)	66 (59, 73)	66 (60, 72)
20-59	495 (24.9%)	80 (27.7%)	206 (23.7%)	119 (25.6%)	90 (24.9%)
60-69	795 (40.1%)	119 (41.2%)	350 (40.2%)	180 (38.8%)	146 (40.4%)
70-79	566 (28.5%)	76 (26.3%)	257 (29.5%)	130 (28.0%)	103 (28.5%)
≥80	129 (6.5%)	14 (4.8%)	58 (6.7%)	35 (7.5%)	22 (6.1%)
Sex					
Female	975 (49.1%)	130 (45.0%)	439 (50.4%)	235 (50.6%)	171 (47.4%)
Male	1,010 (50.9%)	159 (55.0%)	432 (49.6%)	229 (49.4%)	190 (52.6%)
Income quintile					
1 (Lowest)	423 (21.3%)	52 (18.0%)	181 (20.8%)	97 (20.9%)	93 (25.8%)
2	452 (22.8%)	60 (20.8%)	192 (22.0%)	116 (25.0%)	84 (23.3%)
3	377 (19.0%)	57 (19.7%)	175 (20.1%)	86 (18.5%)	59 (16.3%)
4	377 (19.0%)	61 (21.1%)	167 (19.2%)	85 (18.3%)	64 (17.7%)
5 (Highest)	351 (17.7%)	58 (20.1%)	154 (17.7%)	79 (17.0%)	60 (16.6%)
Urban/rural residence					
Urban (RIO<10)	1,223 (61.6%)	193 (66.8%)	537 (61.7%)	278 (59.9%)	215 (59.6%)
Suburban (10≤RIO<40)	518 (26.1%)	60 (20.8%)	234 (26.9%)	123 (26.5%)	101 (28.0%)
Rural (RIO≥40)	207 (10.4%)	29 (10.0%)	87 (10.0%)	57 (12.3%)	34 (9.4%)
Chronic conditions[b]					
Asthma	238 (12.0%)	30 (10.4%)	107 (12.3%)	55 (11.9%)	46 (12.7%)
COPD	705 (35.5%)	99 (34.3%)	289 (33.2%)	160 (34.5%)	157 (43.5%)
Hypertension	1,028 (51.8%)	145 (50.2%)	459 (52.7%)	231 (49.8%)	193 (53.5%)
CHF	121 (6.1%)	16 (5.5%)	58 (6.7%)	24 (5.2%)	23 (6.4%)
Dementia	14 (0.7%)	≤5	≤5	6 (1.3%)	≤5
CKD	94 (4.7%)	12 (4.15%)	35 (4.0%)	24 (5.2%)	23 (6.4%)
**Clinical Characteristics**					
Histology/morphology					
Neoplasms, NOS	386 (19.4%)	67 (23.2%)	157 (18.0%)	100 (21.6%)	62 (17.2%)
Squamous cell neoplasms	256 (12.9%)	40 (13.8%)	101 (11.6%)	55 (11.9%)	60 (16.6%)
Adenomas or adeno-carcinomas	1,270 (64.0%)	171 (59.2%)	590 (67.7%)	292 (62.9%)	217 (60.1%)
Other	73 (3.7%)	11 (3.8%)	23 (2.6%)	17 (3.7%)	22 (6.1%)
ESAS assessment[c]					
Number of assessments					
N	1,723 (86.8%)	250 (86.5%)	765 (87.8%)	422 (90.9%)	286 (79.2%)
Median (IQR)	4 (2, 5)	4 (2, 6)	4 (2, 6)	3 (2, 5)	3 (2, 5)

© 2024 Gupta A et al. *JAMA Network Open.*

Localized physical symptom score					
Median (IQR)	6 (4, 8)	7 (4, 8)	7 (4, 8)	6 (4, 8)	6 (4, 8)
Generalized physical symptom score					
Median (IQR)	7 (5, 9)	8 (6, 9)	7 (5, 9)	7 (5, 9)	7 (5, 9)
Mood-based symptom score					
Median (IQR)	5 (2, 7)	5 (3, 7)	5 (2, 7)	4 (2, 7)	4 (2, 7)
Nearest cancer center from place of residence[d]					
Estimated shortest driving distance (in kilometers)					
Median (IQR)	23 (9, 57)	22 (9, 50)	23 (10, 58)	23 (9, 58)	24 (9, 57)
Estimated shortest driving duration (in minutes)					
Median (IQR)	26 (15, 48)	25 (14, 46)	26 (15, 48)	25 (15, 47)	25 (14, 48)

Acronyms:
IQR, interquartile range; RIO, Rurality Index for Ontario; COPD, chronic obstructive pulmonary disease; CHF, congestive heart failure; CKD, chronic kidney disease; NOS, not otherwise specified; ESAS, Edmonton Symptom Assessment System

Notes:

a. Column percentages may not sum to 100% due to missing data. Small cell responses with ≤5 patients were suppressed.
b. Chronic kidney disease (CKD) was measured with an average estimated glomerular filtration rate (eGFR) of <60 mL/min/1.73 m^2 from multiple laboratory tests within a 1-year lookback period from NSCLC diagnosis.
c. ESAS assessments were measured within a 3-month lookback and lookforward periods from NSCLC diagnosis; ESAS symptoms were categorized as localized physical (pain, nausea and shortness of breath), generalized physical (tiredness, drowsiness, lack of appetite and wellbeing) and mood-based symptom clusters (anxiety and depression); maximum intensity scores from any ESAS symptom within the cluster were used when multiple assessments were reported.
d. Driving distance and duration were measured with the shortest distance generated from the Open Source Routing Machine (OSRM) API with OpenStreetMap data between the postal code of residence and the geographic location of the regional cancer center.

© 2024 Gupta A et al. *JAMA Network Open.*

eTable 7: Overall survival and contact days for patients diagnosed with stage IV non-small cell lung cancer (NSCLC) from January 1, 2014, to December 31, 2017, in Ontario, Canada, stratified by overall survival and time from diagnosis to systemic therapy initiation

**Overall Survival**				
	**≤6 Months (N=3,844)**	**>6 Months (N=1,941)**		
Median (IQR) overall survival	63 (35, 108) days	332 (245, 468) days		
Median (IQR) contact days	27 (17, 41) days	68 (48, 96) days		
Median percentage of contact days	42.9%	20.5%		
**Time from NSCLC Diagnosis to Systemic Therapy Initiation**				
	**≤1 Month (N=289)**	**>1-to-2 Months (N=871)**	**>2-to-3 Months (N=464)**	**>3 Months (N=361)**
Median (IQR) overall survival	211 (103, 346) days	249 (135, 420) days	249 (156.5, 403.5) days	339 (235, 480) days
Median (IQR) contact days	49 (32, 77) days	58 (41, 87) days	56.5 (40, 88) days	69 (54, 96) days
Median percentage of contact days	23.2%	23.3%	22.7%	20.4%

Acronyms:
IQR, interquartile range

© 2024 Gupta A et al. *JAMA Network Open.*

eTable 8: Multivariable analyses for healthcare contact days in specific months for patients diagnosed with stage IV non-small cell lung cancer (NSCLC) from January 1, 2014, to December 31, 2017, and not receiving systemic therapy from January 1, 2014, to December 31, 2019, in Ontario, Canada

Patient Characteristics	First Month		Lowest Contact Month		Last Month	
	ARR (95% CI)	P-value	ARR (95% CI)	P-value	ARR (95% CI)	P-value
**Sociodemographic Characteristics[a]**						
Age						
20-59 (Reference)						
60-69	1.02 (0.96, 1.08)	0.490	1.00 (0.90, 1.12)	0.929	0.98 (0.91, 1.06)	0.608
70-79	1.00 (0.94, 1.06)	0.938	0.91 (0.82, 1.02)	0.118	0.95 (0.88, 1.03)	0.247
≥80	0.93 (0.87, 0.99)	0.022	0.72 (0.64, 0.82)	<0.001	0.86 (0.79, 0.94)	0.001
Sex						
Female (Reference)						
Male	1.00 (0.96, 1.04)	0.967	1.10 (1.02, 1.17)	0.011	1.03 (0.99, 1.08)	0.170
Income quintile						
1 (Lowest) (Reference)						
2	0.93 (0.89, 0.98)	0.006	0.94 (0.85, 1.03)	0.184	0.95 (0.89, 1.01)	0.125
3	0.97 (0.92, 1.02)	0.279	0.99 (0.90, 1.10)	0.874	0.92 (0.86, 0.99)	0.028
4	0.94 (0.89, 0.99)	0.026	1.05 (0.94, 1.17)	0.381	1.02 (0.94, 1.09)	0.661
5 (Highest)	0.94 (0.88, 0.99)	0.032	0.98 (0.88, 1.10)	0.770	0.94 (0.87, 1.02)	0.137
Urban/rural residence						
Urban (RIO<10)	1.10 (1.01, 1.21)	0.039	1.18 (0.98, 1.42)	0.075	1.06 (0.94, 1.20)	0.374
Suburban (10≤RIO<40)	1.03 (0.95, 1.11)	0.544	1.12 (0.95, 1.31)	0.168	1.03 (0.93, 1.15)	0.567
Rural (RIO≥40) (Reference)						
Chronic conditions[b]						
Asthma (Yes vs. No)	0.99 (0.94, 1.04)	0.694	1.00 (0.90, 1.11)	0.969	1.00 (0.93, 1.08)	0.952
COPD (Yes vs. No)	0.97 (0.93, 1.01)	0.115	0.94 (0.87, 1.01)	0.101	0.95 (0.91, 1.00)	0.057
Hypertension (Yes vs. No)	0.98 (0.94, 1.02)	0.264	0.96 (0.88, 1.03)	0.265	1.01 (0.95, 1.06)	0.846
CHF (Yes vs. No)	1.00 (0.94, 1.07)	0.943	1.06 (0.94, 1.19)	0.350	1.05 (0.97, 1.13)	0.194
Dementia (Yes vs. No)	1.13 (1.04, 1.23)	0.006	1.06 (0.91, 1.25)	0.446	1.12 (1.00, 1.24)	0.045
CKD (Yes vs. No)	1.01 (0.95, 1.07)	0.866	1.03 (0.92, 1.15)	0.587	1.03 (0.96, 1.11)	0.395
**Clinical Characteristics[a]**						
ESAS assessment[c] (Yes vs. No)	0.61 (0.58, 0.63)	<0.001	0.39 (0.36, 0.43)	<0.001	0.66 (0.62, 0.70)	<0.001
Nearest cancer center from place of residence[d]						
Estimated shortest driving distance (Per 25-km increase)	0.99 (0.98, 1.01)	0.340	1.01 (0.98, 1.03)	0.591	1.01 (0.99, 1.03)	0.242

Acronyms:

© 2024 Gupta A et al. *JAMA Network Open.*

ARR, adjusted relative risk; CI, confidence interval; RIO, Rurality Index for Ontario; COPD, chronic obstructive pulmonary disease; CHF, congestive heart failure; CKD, chronic kidney disease; ESAS, Edmonton Symptom Assessment System; km, kilometer

Notes:

a. Additional sociodemographic and clinical characteristics included (but not shown) in the multivariable analyses: Place of residence, smoking status, Elixhauser comorbidity index, histology/morphology, anatomical location, palliative radiotherapy, metastasis surgery, year of diagnosis, inpatient hospitalizations and emergency department (ED)-only visits.
b. Chronic kidney disease (CKD) was measured with an average estimated glomerular filtration rate (eGFR) of <60 mL/min/1.73 m^2 from multiple laboratory tests within a 1-year lookback period from NSCLC diagnosis.
c. ESAS assessments were measured within a 3-month lookback and lookforward periods from NSCLC diagnosis.
d. Driving distance was measured with the shortest distance generated from the Open Source Routing Machine (OSRM) API with OpenStreetMap data between the postal code of residence and the geographic location of the regional cancer center.

© 2024 Gupta A et al. *JAMA Network Open.*

eTable 9: Multivariable analyses for healthcare contact days in specific months for patients diagnosed with stage IV non-small cell lung cancer (NSCLC) from January 1, 2014, to December 31, 2017, and receiving systemic therapy from January 1, 2014, to December 31, 2019, in Ontario, Canada

Patient Characteristics	First Month		Lowest Contact Month		Last Month	
	ARR (95% CI)	P-value	ARR (95% CI)	P-value	ARR (95% CI)	P-value
**Sociodemographic Characteristics[a]**						
Age						
20-59 (Reference)						
60-69	0.93 (0.88, 0.99)	0.027	0.86 (0.74, 0.99)	0.035	1.02 (0.92, 1.13)	0.714
70-79	0.89 (0.83, 0.95)	<0.001	0.81 (0.68, 0.95)	0.010	0.97 (0.86, 1.10)	0.677
≥80	0.86 (0.76, 0.96)	0.011	0.68 (0.48, 0.95)	0.025	0.82 (0.66, 1.01)	0.063
Sex						
Female (Reference)						
Male	1.00 (0.96, 1.05)	0.923	1.00 (0.89, 1.12)	0.980	0.92 (0.85, 1.00)	0.052
Income quintile						
1 (Lowest) (Reference)						
2	1.03 (0.96, 1.10)	0.474	1.22 (1.02, 1.46)	0.030	0.99 (0.88, 1.12)	0.924
3	1.12 (1.04, 1.21)	0.003	1.26 (1.06, 1.49)	0.010	1.00 (0.88, 1.13)	0.980
4	1.08 (0.99, 1.16)	0.068	1.21 (1.01, 1.45)	0.040	1.06 (0.94, 1.20)	0.327
5 (Highest)	1.05 (0.97, 1.13)	0.238	1.21 (1.01, 1.46)	0.043	0.87 (0.76, 1.00)	0.044
Urban/rural residence						
Urban (RIO<10)	1.07 (0.94, 1.21)	0.311	1.10 (0.82, 1.47)	0.539	1.33 (1.10, 1.62)	0.004
Suburban (10≤RIO<40)	1.03 (0.92, 1.15)	0.606	1.21 (0.94, 1.56)	0.141	1.16 (0.97, 1.38)	0.098
Rural (RIO≥40) (Reference)						
Chronic conditions[b]						
Asthma (Yes vs. No)	0.99 (0.92, 1.06)	0.763	1.03 (0.89, 1.20)	0.675	0.94 (0.83, 1.07)	0.362
COPD (Yes vs. No)	0.93 (0.88, 0.98)	0.004	0.94 (0.83, 1.06)	0.309	1.03 (0.94, 1.13)	0.495
Hypertension (Yes vs. No)	1.00 (0.95, 1.05)	0.979	1.03 (0.91, 1.16)	0.681	1.03 (0.94, 1.13)	0.515
CHF (Yes vs. No)	1.13 (1.01, 1.26)	0.032	1.09 (0.85, 1.39)	0.507	1.07 (0.91, 1.25)	0.413
Dementia (Yes vs. No)	1.22 (0.78, 1.92)	0.384	0.97 (0.41, 2.26)	0.939	1.35 (0.89, 2.05)	0.154
CKD (Yes vs. No)	1.00 (0.89, 1.11)	0.936	1.12 (0.88, 1.44)	0.357	0.87 (0.71, 1.06)	0.157
**Clinical Characteristics[a]**						
Palliative systemic therapy[e]						
Number of lines of systemic therapy						
1 (Reference)						
2	0.92 (0.86, 0.98)	0.007	0.64 (0.55, 0.75)	<0.001	0.89 (0.80, 0.99)	0.036
≥3	0.84 (0.78, 0.92)	<0.001	0.45 (0.37, 0.54)	<0.001	0.97 (0.84, 1.12)	0.665

© 2024 Gupta A et al. *JAMA Network Open.*

Regimen						
Only cytotoxic, or trial/other therapy (Reference)						
1st/subsequent-line immunotherapy with/without chemotherapy	1.02 (0.94, 1.10)	0.670	0.94 (0.80, 1.12)	0.492	0.91 (0.80, 1.04)	0.177
1st/subsequent-line targeted therapy	1.09 (1.02, 1.17)	0.014	0.78 (0.65, 0.93)	0.007	0.99 (0.89, 1.11)	0.885
ESAS assessment[c] (Yes vs. No)	0.89 (0.82, 0.97)	0.010	0.87 (0.71, 1.06)	0.163	1.02 (0.90, 1.15)	0.791
Nearest cancer center from place of residence[d]						
Estimated shortest driving distance (Per 25-km increase)	0.99 (0.97, 1.01)	0.245	0.99 (0.96, 1.03)	0.608	1.03 (1.01, 1.05)	0.005

Acronyms:
ARR, adjusted relative risk; CI, confidence interval; RIO, Rurality Index for Ontario; COPD, chronic obstructive pulmonary disease; CHF, congestive heart failure; CKD, chronic kidney disease; ESAS, Edmonton Symptom Assessment System; km, kilometer

Notes:

a. Additional sociodemographic and clinical characteristics included (but not shown) in the multivariable analyses: Place of residence, smoking status, Elixhauser comorbidity index, histology/morphology, anatomical location, palliative radiotherapy, metastasis surgery, year of diagnosis, inpatient hospitalizations and emergency department (ED)-only visits.
b. Chronic kidney disease (CKD) was measured with an average estimated glomerular filtration rate (eGFR) of <60 mL/min/1.73 m^2 from multiple laboratory tests within a 1-year lookback period from NSCLC diagnosis.
c. ESAS assessments were measured within a 3-month lookback and lookforward periods from NSCLC diagnosis.
d. Driving distance was measured with the shortest distance generated from the Open Source Routing Machine (OSRM) API with OpenStreetMap data between the postal code of residence and the geographic location of the regional cancer center.

© 2024 Gupta A et al. *JAMA Network Open.*

eTable 10: Multivariable analyses for healthcare contact days in specific months for patients diagnosed with stage IV non-small cell lung cancer (NSCLC) from January 1, 2014, to December 31, 2017, receiving systemic therapy from January 1, 2014, to December 31, 2019, and completing ESAS assessments in Ontario, Canada

**Patient Characteristics**	**First Month**		**Lowest Contact Month**		**Last Month**	
	**ARR (95% CI)**	**P-value**	**ARR (95% CI)**	**P-value**	**ARR (95% CI)**	**P-value**
**Sociodemographic Characteristics[a]**						
Age						
20-59 (Reference)						
60-69	0.92 (0.86, 0.98)	0.010	0.87 (0.75, 1.00)	0.050	0.97 (0.87, 1.09)	0.625
70-79	0.89 (0.83, 0.96)	0.002	0.85 (0.72, 1.00)	0.052	0.95 (0.83, 1.08)	0.415
≥80	0.83 (0.73, 0.94)	0.004	0.68 (0.47, 0.98)	0.039	0.76 (0.59, 0.97)	0.031
Sex						
Female (Reference)						
Male	1.01 (0.96, 1.06)	0.631	1.05 (0.93, 1.18)	0.473	0.91 (0.83, 0.99)	0.037
Income quintile						
1 (Lowest) (Reference)						
2	1.07 (0.99, 1.15)	0.082	1.25 (1.04, 1.50)	0.015	0.99 (0.87, 1.12)	0.880
3	1.16 (1.07, 1.25)	<0.001	1.27 (1.06, 1.51)	0.009	0.98 (0.86, 1.13)	0.810
4	1.12 (1.03, 1.21)	0.008	1.22 (1.01, 1.47)	0.037	1.10 (0.96, 1.25)	0.164
5 (Highest)	1.07 (0.99, 1.16)	0.069	1.25 (1.04, 1.51)	0.017	0.87 (0.75, 1.01)	0.060
Urban/rural residence						
Urban (RIO<10)	1.14 (1.01, 1.29)	0.032	1.32 (1.00, 1.73)	0.049	1.45 (1.18, 1.78)	<0.001
Suburban (10≤RIO<40)	1.09 (0.98, 1.21)	0.133	1.44 (1.14, 1.83)	0.002	1.21 (1.00, 1.46)	0.045
Rural (RIO≥40) (Reference)						
Chronic conditions[d]						
Asthma (Yes vs. No)	1.02 (0.95, 1.09)	0.604	1.06 (0.90, 1.24)	0.490	0.96 (0.84, 1.09)	0.503
COPD (Yes vs. No)	0.92 (0.87, 0.97)	0.002	0.95 (0.84, 1.09)	0.467	1.04 (0.94, 1.14)	0.467
Hypertension (Yes vs. No)	0.99 (0.93, 1.04)	0.577	0.99 (0.88, 1.12)	0.909	1.03 (0.93, 1.13)	0.599
CHF (Yes vs. No)	1.15 (1.03, 1.29)	0.017	0.96 (0.78, 1.18)	0.708	1.01 (0.84, 1.22)	0.900
Dementia (Yes vs. No)	1.19 (0.72, 1.96)	0.497	0.78 (0.39, 1.57)	0.488	1.27 (0.74, 2.18)	0.383
CKD (Yes vs. No)	1.05 (0.94, 1.17)	0.430	1.23 (0.96, 1.58)	0.098	0.91 (0.74, 1.12)	0.368
**Clinical Characteristics[a]**						
Palliative systemic therapy[e]						
Number of lines of systemic therapy						
1 (Reference)						
2	0.94 (0.89, 1.01)	0.085	0.65 (0.56, 0.76)	<0.001	0.86 (0.77, 0.97)	0.013
≥3	0.90 (0.82, 0.98)	0.014	0.46 (0.38, 0.57)	<0.001	0.97 (0.83, 1.13)	0.671

© 2024 Gupta A et al. *JAMA Network Open.*

Regimen						
Only cytotoxic, or trial/other therapy (Reference)						
1st/subsequent-line immunotherapy with/without chemotherapy	1.02 (0.94, 1.10)	0.619	1.02 (0.85, 1.22)	0.863	0.93 (0.81, 1.08)	0.333
1st/subsequent-line targeted therapy	1.07 (1.00, 1.15)	0.052	0.76 (0.63, 0.93)	0.007	0.98 (0.86, 1.10)	0.691
ESAS assessment[f]						
Number of assessments (Per 1-assessment increase)	1.00 (0.99, 1.00)	0.362	0.97 (0.94, 0.99)	0.010	0.99 (0.97, 1.01)	0.457
Localized physical symptom score						
0-3 (Reference)						
4-6	0.92 (0.85, 1.00)	0.045	0.99 (0.83, 1.18)	0.921	1.08 (0.94, 1.24)	0.256
7-10	0.96 (0.89, 1.04)	0.326	1.18 (1.00, 1.40)	0.052	1.00 (0.87, 1.15)	0.977
Generalized physical symptom score						
0-3 (Reference)						
4-6	1.12 (1.02, 1.24)	0.017	1.08 (0.88, 1.33)	0.458	1.07 (0.92, 1.25)	0.365
7-10	1.18 (1.07, 1.30)	<0.001	1.10 (0.90, 1.36)	0.350	1.02 (0.87, 1.20)	0.815
Mood-based symptom score						
0-3 (Reference)						
4-6	0.97 (0.91, 1.03)	0.324	1.14 (0.99, 1.31)	0.062	0.92 (0.82, 1.03)	0.138
7-10	0.97 (0.91, 1.04)	0.375	1.22 (1.05, 1.42)	0.009	0.89 (0.79, 0.99)	0.036
Nearest cancer center from place of residence[h]						
Estimated shortest driving distance (Per 25-km increase)	1.00 (0.98, 1.02)	0.918	1.01 (0.98, 1.04)	0.533	1.04 (1.02, 1.07)	<0.001

Acronyms:
ARR, adjusted relative risk; CI, confidence interval; RIO, Rurality Index for Ontario; COPD, chronic obstructive pulmonary disease; CHF, congestive heart failure; CKD, chronic kidney disease; ESAS, Edmonton Symptom Assessment System; km, kilometer

Notes:

a. Additional sociodemographic and clinical characteristics included (but not shown) in the multivariable analyses: Place of residence, smoking status, Elixhauser comorbidity index, histology/morphology, anatomical location, palliative radiotherapy, metastasis surgery, year of diagnosis, inpatient hospitalizations and emergency department (ED)-only visits.
b. Chronic kidney disease (CKD) was measured with an average estimated glomerular filtration rate (eGFR) of <60 mL/min/1.73 m^2 from multiple laboratory tests within a 1-year lookback period from NSCLC diagnosis.
c. ESAS assessments were measured within a 3-month lookback and lookforward periods from NSCLC diagnosis; ESAS symptoms were categorized as localized physical (pain, nausea and shortness of breath), generalized physical (tiredness, drowsiness, lack of appetite and wellbeing) and mood-based symptom clusters (anxiety and depression); maximum intensity scores from any ESAS symptom within the cluster were used when multiple assessments were reported.

© 2024 Gupta A et al. *JAMA Network Open.*

d. Driving distance was measured with the shortest distance generated from the Open Source Routing Machine (OSRM) API with OpenStreetMap data between the postal code of residence and the geographic location of the regional cancer center.

© 2024 Gupta A et al. *JAMA Network Open.*

eFigure 1: Cohort creation of patients diagnosed with stage IV non-small cell lung cancer (NSCLC) from January 1, 2014, to December 31, 2017, in Ontario, Canada

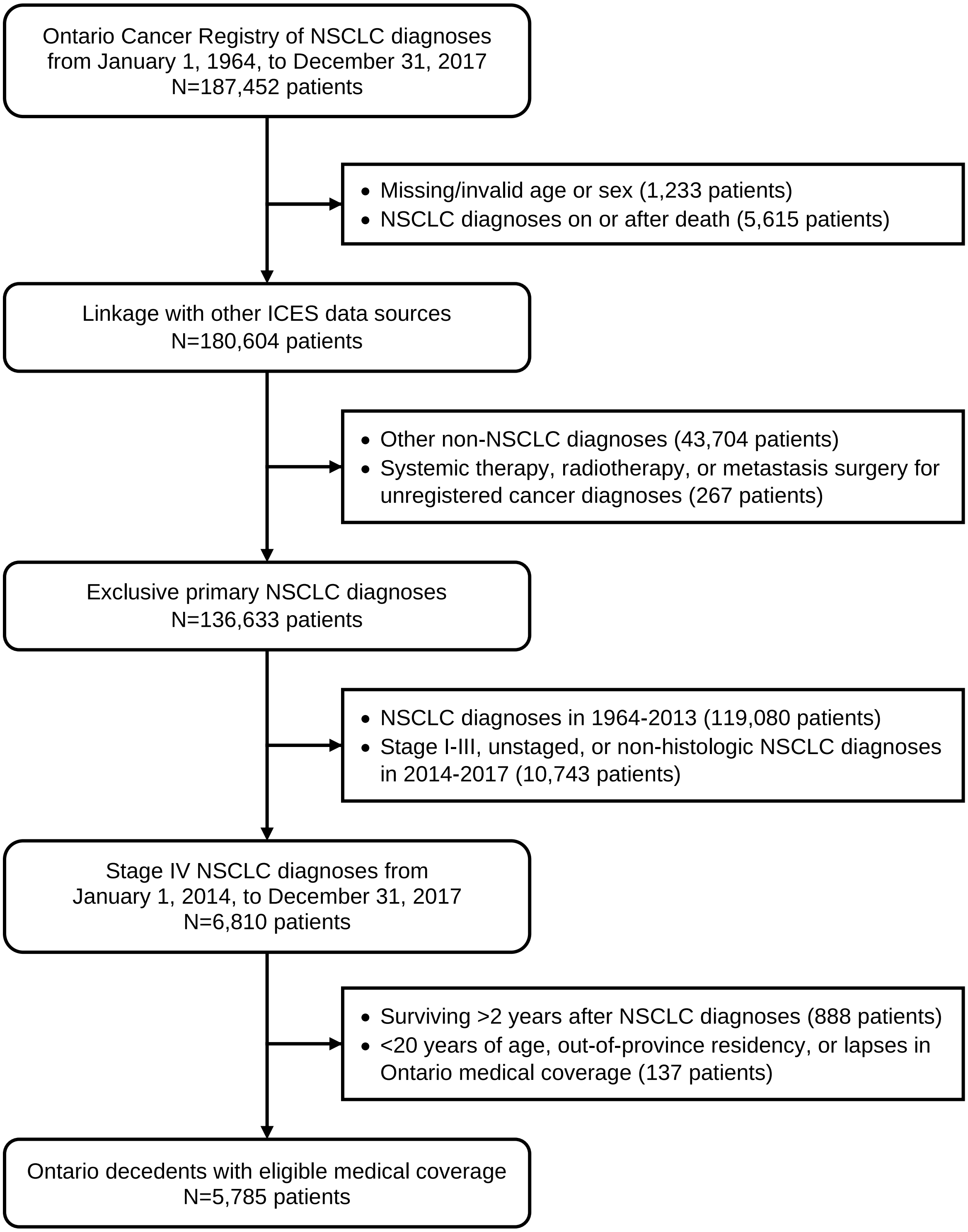

© 2024 Gupta A et al. *JAMA Network Open.*

eFigure 2: Weekly contact days, stratified by survival, for patients diagnosed with stage IV non-small cell lung cancer (NSCLC) from January 1, 2014, to December 31, 2017, in Ontario, Canada

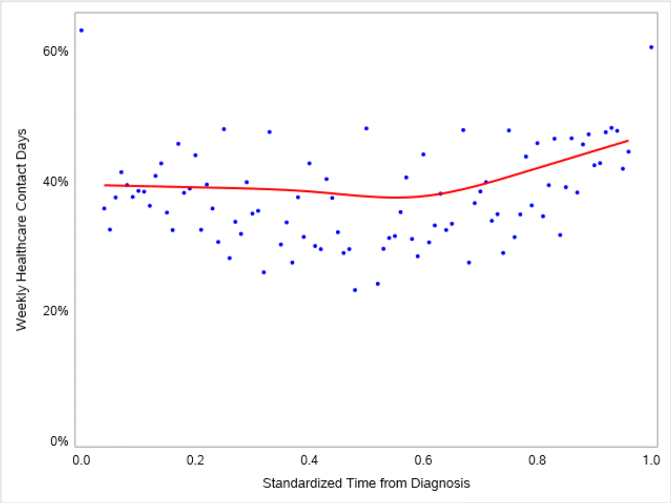

(A) Total contact days for patients with ≤6 months of survival

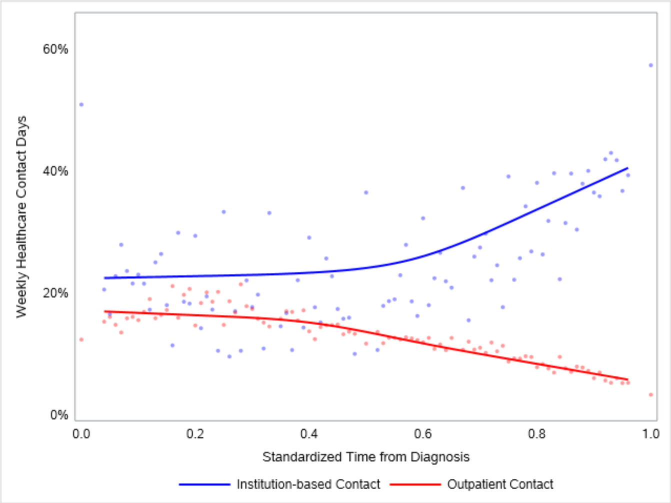

(B) Institution-based and outpatient contact days for patients with ≤6 months of survival

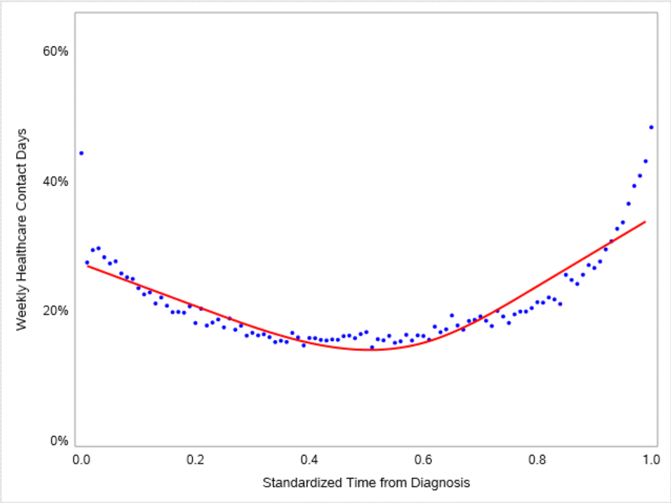

(C) Total contact days for patients with >6 months of survival

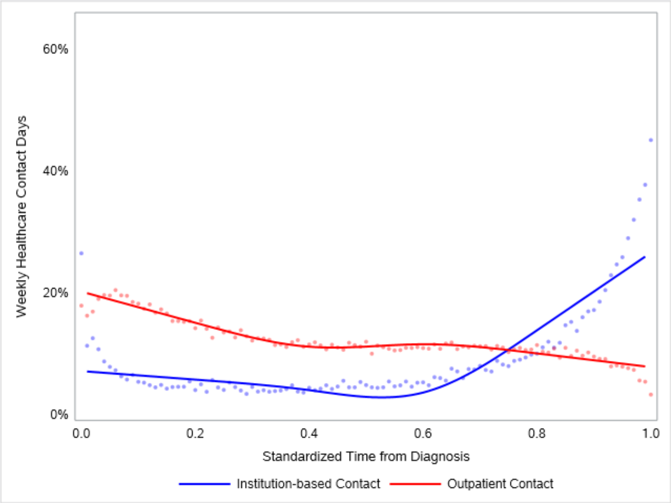

(D) Institution-based and outpatient contact days for patients with >6 months of survival

© 2024 Gupta A et al. *JAMA Network Open.*

eFigure 3: Weekly contact days, stratified by time to initiation of systemic therapy, for patients diagnosed with stage IV non-small cell lung cancer (NSCLC) from January 1, 2014, to December 31, 2017, in Ontario, Canada

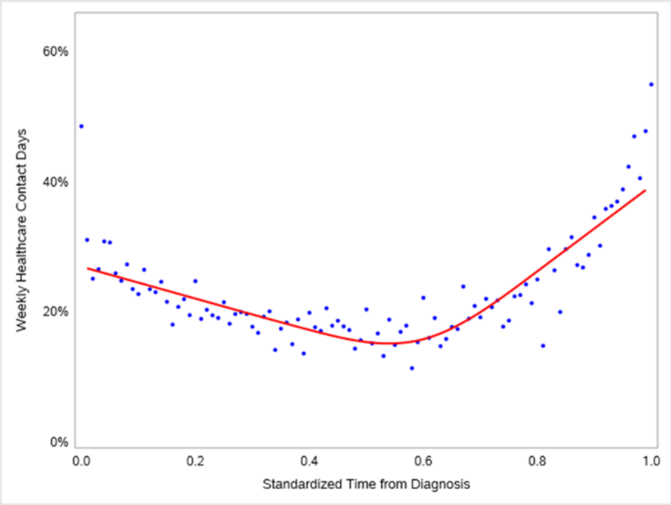

(A) Total contact days for patients who initiated systemic therapy with ≤1 month from NSCLC diagnosis

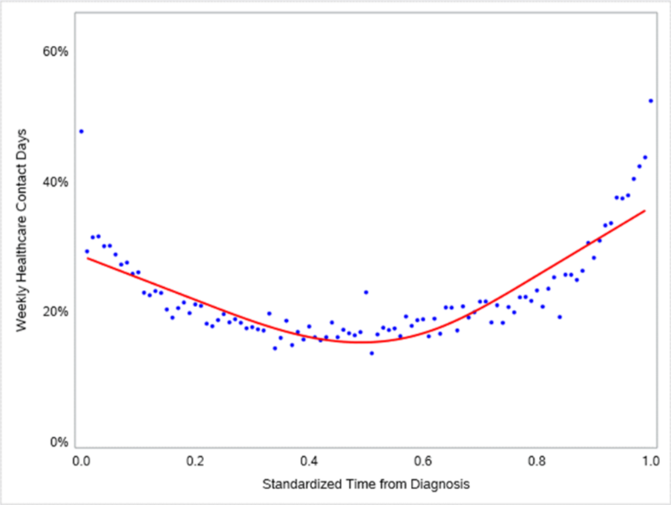

(B) Total contact days for patients who initiated systemic therapy with >1-to-2 months from NSCLC diagnosis

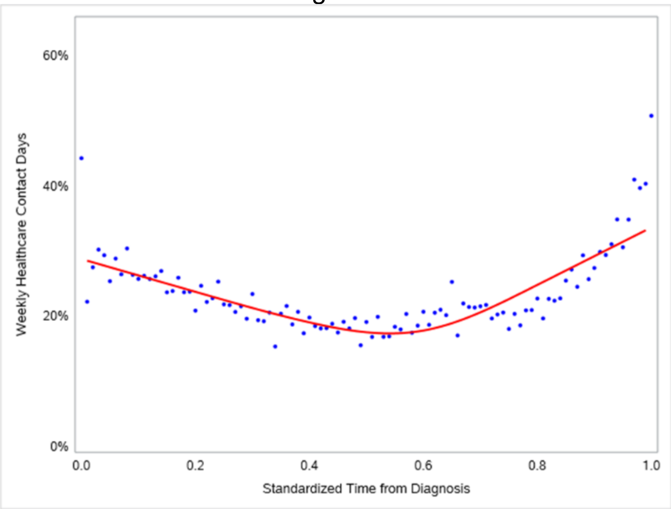

(C) Total contact days for patients who initiated systemic therapy with >2-to-3 months from NSCLC diagnosis

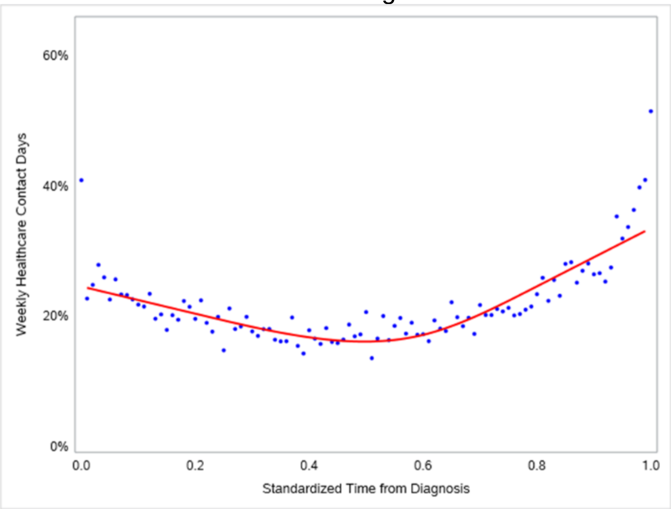

(D) Total contact days for patients who initiated systemic therapy with >3 months from NSCLC diagnosis

© 2024 Gupta A et al. *JAMA Network Open.*
