# Supplementary material for: Trajectories of Health Care Contact Days for Patients With Stage IV Non–Small Cell Lung Cancer
Source: JAMA Netw Open. 2024 Apr 8;7(4):e244278. doi: 10.1001/jamanetworkopen.2024.4278 (PMC11002696; doi:10.1001/jamanetworkopen.2024.4278)
Supplement: Supplement 2. — Data Sharing Statement [file jamanetwopen-e244278-s002.pdf]

Data Sharing Statement

Gupta. Trajectories of Health Care Contact Days for Patients With Stage IV Non–Small Cell Lung Cancer. *JAMA Netw Open*. Published April 08, 2024.
doi:10.1001/jamanetworkopen.2024.4278

Data
Data available: No

Additional Information
Explanation for why data not available: The full dataset from this study is held securely in coded form at ICES. While data sharing agreements prohibit ICES from making the dataset publicly available, access may be granted to those who meet pre-specified criteria for confidential access, available at www.ices.on.ca/DAS. The full dataset creation plan and underlying analytic code are available from the authors upon request, understanding that the computer programs may rely upon coding templates or macros that are unique to ICES and therefore may require modification.
